# The Kappa Opioid Receptor: A Promising Therapeutic Target for Multiple Pathologies

**DOI:** 10.3389/fphar.2022.837671

**Published:** 2022-06-20

**Authors:** Martin L. Dalefield, Brittany Scouller, Rabia Bibi, Bronwyn M. Kivell

**Affiliations:** Centre for Biodiscovery, School of Biological Sciences, Victoria University of Wellington, Wellington, New Zealand

**Keywords:** kappa opioid agonist, clinical trials, pharmacotherapies, drug-development, multiple sclerosis, pain, biased agonist, pruritis

## Abstract

Kappa-opioid receptors (KOR) are widely expressed throughout the central nervous system, where they modulate a range of physiological processes depending on their location, including stress, mood, reward, pain, inflammation, and remyelination. However, clinical use of KOR agonists is limited by adverse effects such as dysphoria, aversion, and sedation. Within the drug-development field KOR agonists have been extensively investigated for the treatment of many centrally mediated nociceptive disorders including pruritis and pain. KOR agonists are potential alternatives to mu-opioid receptor (MOR) agonists for the treatment of pain due to their anti-nociceptive effects, lack of abuse potential, and reduced respiratory depressive effects, however, dysphoric side-effects have limited their widespread clinical use. Other diseases for which KOR agonists hold promising therapeutic potential include pruritis, multiple sclerosis, Alzheimer’s disease, inflammatory diseases, gastrointestinal diseases, cancer, and ischemia. This review highlights recent drug-development efforts targeting KOR, including the development of G-protein–biased ligands, mixed opioid agonists, and peripherally restricted ligands to reduce side-effects. We also highlight the current KOR agonists that are in preclinical development or undergoing clinical trials.

## 1 Introduction

The endogenous opioid system consists of a family of peptides that include β-endorphin, the enkephalins, and dynorphins. These endogenous ligands bind to mu opioid receptors (MOR), delta opioid receptors (DOR), kappa opioid receptors (KOR) and opioid receptor–like 1 receptors (NOP) (Kieffer, 1995), which belong to the rhodopsin family of G-protein coupled receptors (GPCRs) (Rogers, 2020). They are widely expressed throughout the central nervous system (CNS) (Mansour et al., 1995) and within the gastrointestinal tract, respiratory system, heart (Holzer, 2009; Peng et al., 2012; Sobanski et al., 2014; Jamshidi et al., 2015), the peripheral terminals of sensory nerves, immune cells, and endocrine glands, where they contribute to various physiological functions such as nociception (Stein et al., 1989; Iwaszkiewicz et al., 2013), gastrointestinal transit (Holzer, 2009), respiration (Zebraski et al., 2000; Pattinson, 2008), endocrine (Vuong et al., 2010; Fountas et al., 2018), and immune functions (Eisenstein, 2019).

Endogenous opioid peptides and their receptors are expressed in limbic and paralimbic regions in human and rodent brains (Kuhar et al., 1973; Delay-Goyet et al., 1987; Bagnol et al., 1995; Peckys and Landwehrmeyer, 1999) and are involved in the modulation of affective states, neuroendocrine and autonomic stress responses, mood and motivational states. The clinically relevant analgesic effects and side effects produced by each member of the endogenous opioid system have been summarized by Günther et al. (2018). MOR is the target of classic opioid analgesics such as morphine. Analgesics acting on MOR are commonly used to treat moderate-to-severe acute pain and cancer pain. However, MOR agonism also causes respiratory depression, tolerance, and dependence and comes with a high risk of addiction (Pasternak and Pan, 2013). Indeed, overreliance on MOR agonists for the treatment of pain has led to a global epidemic of opioid abuse, with MOR agonist prescriptions serving as a gateway to illicit drug use.

Although MOR agonists are effective at treating acute pain, they are largely ineffective when used long-term because they induce hyperalgesia and tolerance (Matthes et al., 1996; Chu et al., 2006; Rowbotham and Wallace, 2020), and, with the exception of treating cancer pain, they are not intended as first-line chronic pain pharmacotherapies. However, they are often used due to lack of effective treatment options available. Chronic pain is persistent pain caused by neuroplastic changes within the CNS and it is estimated that 20% of adults worldwide suffer from chronic pain (Goldberg and McGee, 2011), while the socioeconomic impact is estimated to be US$635 billion annually (Institute of Medicine, 2011). The result has been escalating numbers of opioid overdose deaths over the last 20 years (Zelaya et al., 2020).

The pain medications market exceeds $80 billion per annum (Allied Market Research, 2020), and there are high levels of interest in the development of novel drugs that provide analgesic effects but do not come with the abuse risks of MOR agonists. The DOR has received some attention as a potential therapeutic target, but DOR agonists can induce epileptic seizures (Chung et al., 2015) and also maladaptively stimulate dopaminergic reward pathways (Suzuki et al., 1996). For a recent review on the role of DOR in modulating nociception see Quirion et al. (2020). Agonists targeting the KOR have also received growing interest (Kivell and Prisinzano, 2010; Bohn and Aubé, 2017; Beck and Dix, 2019; Mores et al., 2019; Paton et al., 2020a; Mercadante and Romualdi, 2020).

KOR agonists play a key role in analgesia and are particularly relevant to peripherally mediated nociceptive disorders such as pruritus (Kumagai et al., 2010; Viscusi et al., 2021). KOR agonists have received attention in the past due to being non-addictive anti-nociceptive drugs that do not induce respiratory depression (Viscusi et al., 2021; Wang et al., 2021), properties that compare favourably with those of MOR agonists such as morphine. However, they have their own distinctive side effect profiles that include stress and aversion (Dykstra et al., 1987; Land et al., 2008; Wee and Koob, 2010), depression (Knoll and Carlezon, 2010), sedation (Dykstra et al., 1987), diuresis (Meariman et al., 2021), and neuroendocrine effects (increase in serum prolactin, cortisol, and adrenocorticotropic hormone levels) (Ko and Husbands, 2020). These side effects have hindered clinical development. In recent years, pharmacologists have turned to strategies that may allow the development of KOR agonists that provide anti-nociception without these side effects. Three strategies that are commonly utilised to reduce side effects are to develop G-protein biased agonists (Mores et al., 2019), mixed opioid agonists (Atigari et al., 2021), or peripherally restricted KOR agonists, because most KOR-mediated side effects are centrally mediated (Machelska and Celik, 2018), with the exception of diuresis which has both peripheral (Salas et al., 1989; Butelman et al., 1999; Albert-Vartanian et al., 2016) and CNS-mediated components (Leander, 1983; Brooks et al., 1993; Kapusta and Obih, 1993).

In this review, we highlight research into the therapeutic potential of KOR agonists within key diseases including nociception (Paton et al., 2017), pruritis (Wang et al., 2021), multiple sclerosis (MS) (Denny et al., 2021), Alzheimer’s disease (AD) (Song et al., 2021), immune mediated diseases such as osteoarthritis (Wilson et al., 1996), atopic dermatitis (Nakasone et al., 2015), food allergy (Duncker et al., 2012), gastrointestinal diseases (Mangel et al., 2008), cancer (Yamamizu et al., 2013), and hypoxia and ischemia (Wu et al., 2021). We also review the pharmacological strategies being used to develop safer, more effective KOR agonists with limited associated side effects.

## 2 Strategies for Developing Better, Safer Kappa-Opioid Receptors Agonists for Therapeutic Use

The canonical KOR signalling pathways includes both G-protein and β-arrestin-2 dependent signalling pathways (Bruchas and Chavkin, 2010). There is growing evidence suggesting that KOR signalling through G-protein pathways mediates the anti-nociceptive and anti-pruritic effects of KOR agonists, whereas β-arrestin-2-dependent signalling mediates the dysphoric effects of KOR agonists (Valentino and Volkow, 2018). Experiments performed in β-arrestin-2 knockout mice have yielded evidence that this pathway is unnecessary for KOR agonists to exert anti-pruritic effects (Morgenweck et al., 2015), and multiple lines of evidence show that β-arrestin-2 signalling and p38 activation induces conditioned place aversion (CPA) (Bruchas and Chavkin, 2010). A recent study of 21 structurally diverse KOR ligands also revealed a correlation between β-arrestin-2 recruitment and sedative effects in mice evaluated with the rotarod test (Dunn et al., 2019). The observed associations between signalling pathways specific behavioural effects have prompted efforts to develop G-protein biased KOR agonists. Utilising this strategy, it may be possible to develop KOR agonists with an improved therapeutic index (Mores et al., 2019). For recent reviews see Bohn and Aubé (2017) and Paton et al. (2020a). It is also possible that low-efficacy partial agonists may be responsible for improved side-effects, as has been found with MOR agonists (Gillis et al., 2020).

Alternative strategies for developing better KOR agonists also include the development of mixed opioid agonists. For example, Uprety et al. (2021) and Atigari et al. (2021) provide evidence that the morphinan analogue MP1104, activates KOR preferentially over DOR, with little effect at MOR *in vivo.* MP1104 produces anti-nociceptive effects in mice and rats without causing respiratory depression, conditioned place preference, or conditioned place aversion, sedation, or cross-tolerance with morphine. This appears to be due to DOR agonism opposing the side-effects of KOR agonism while potentiating anti-nociceptive effects.

Recently, there has been considerable success in the development of peripherally restricted KOR agonists. By reducing the CNS penetrance of KOR agonists, it is possible to reduce centrally mediated side effects. This is typically done by performing structural modifications to increase hydrophilicity or use of nanocarrier-based approaches or synthesis of peptide-based compounds (Machelska and Celik, 2018). The potential drawback to this strategy is that it limits the therapeutic actions of KOR to the periphery and is unlikely to be successful in targeting diseases of the CNS. Successful approaches using peripherally restricted KOR agonists have been shown to attenuate nociceptive transmission by acting on KORs located in the viscera, and such drugs have been proven clinically effective in reducing pruritis without causing centrally mediated side effects. This approach has recently yielded a new US Food and Drug Administration (FDA)-approved drug (Korsuva, also known as CR845 and difelikefalin) to treat pruritis (Deeks, 2021). See Section 3.2 for further information.

Examples utilising these strategies are presented within Tables 1, 2 and within each section on specific diseases where KOR agonism may hold therapeutic potential.

**TABLE 1:** Therapeutic effects and side effects of KOR agonists in preclinical studies.

KOR agonist	Pre-clinical therapeutic effects	Side effects	References
Family: Arylacetamides			
ADL 10-0101 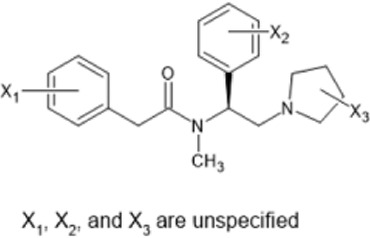	↓ Nociception (8.8 mg/kg, s.c.) inflammatory model (rat, mouse) [1]	ND	Gottshall et al. (1999) [1]; Sandner-Kiesling et al. (2002) [2]
	↓ Nociception: Formalin model (8.8 mg/kg, s.c., rat) [2]		
	↓ Writhing (0.018 mg/kg, s.c.) acetic acid-induced writhing (mice) [1]		
	↓ Hyperalgesia (8.8 mg/kg, s.c.) Freund’s complete adjuvant model (mice) [1]		
Asimadoline 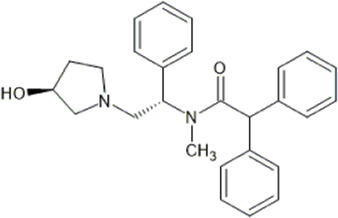	↓ Nociception: formalin test (phase I ID_50_ 1.9 mg/kg, s.c., and 10.4 mg/kg, p.o.; phase II ID_50_ 0.26 mg/kg, s.c., and 3.5 mg/kg, p.o. mouse, M) and abdominal constriction test (ID50 mouse 1.75 mg/kg, s.c., and 8.4 mg/kg, p.o.; ID_50_ rat 3.2 mg/kg, s.c., and 250 mg/kg, p.o.)[3]	↑ Diuresis (1 mg/kg, s.c. and 10 mg/kg, p.o., rat) [3]	Barber et al. (1994) [3]; Caram-Salas et al. (2007) [4]; Marsella et al. (2021) [5]
	↓ Allodynia: spinal nerve ligation (1–30 mg/kg, s.c., rat, M & F) [4]		
	↓ Severity of dermatitis [1% asimadoline gel) canine atopic dermatitis model (topical application] [5]		
BRL-52537 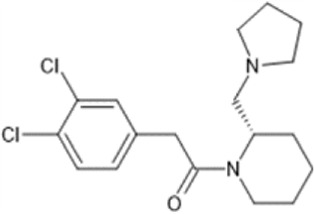	↑ Neuroprotection: focal cerebral ischemia (1 mg/kg/hour, i.v., rat, M) [6]	↓ Motor activity (0.1 mg/kg, i.p., M) [7]	Goyagi et al. (2003) [6]; Kuzmin et al. (2000) [7]; Peart et al. (2004) [8]; Jung et al. (2017) [9]; Fang et al. (2013) [10]; Shahbazian et al. (2002) [11]
	↓ Arrhythmia: Ischemia Model (0.5 mg/kg, p.o., rat, M) [8]		
	↓ Allodynia: Neuropathic spinal nerve ligation (SNL) (ED50 0.71 μg/kg, i.t., in rat, M) [9]		
	↓Neuronal apoptosis and in cerebral ischemia/reperfusion injury (1 mg/kg/h, i.v., rat, M) [10]		
	↓ Intestinal peristalsis *ex vivo* guinea-pig small intestine model (0.3–44.3 nM, Organ bath) [11]		
Enadoline 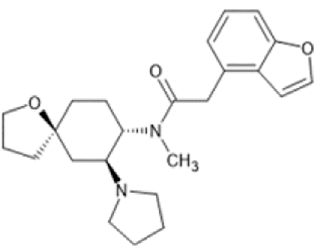	↓ Thermal hyperalgesia: plantar test (1–100 μg/kg, i.v., rat, M) [12]	Mild sedation (25–100 g/kg, i.v.) [13]	Field et al. (1999) [12]; Tortella et al. (1997) [13]; Hughes et al. (1998) [14]
	↓ Allodynia (mechanical) (1–100 μg/kg, i.v., rat, M) [12]		
	↓ Levodopa-induced dyskinesia parkinsonism (100 mg/kg, i.p., rat, M) [14]		
Fedotozine 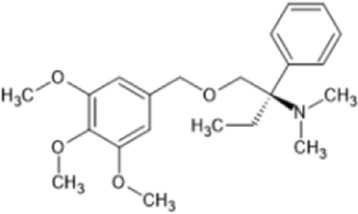	↓ Peritoneal irritation: acetic acid model (2.4 mg/kg s.c., rat, M) [15]	n/e diuresis (0.1–30 mg/kg, s.c) [16]	Riviere et al. (1994) [15]; Soulard et al. (1996) [16]; Bonaz et al. (2000) [17]
	↓ Visceral nociception: acetic acid induced (15 mg/kg, s.c., rat, M) [17]		
GR-89696 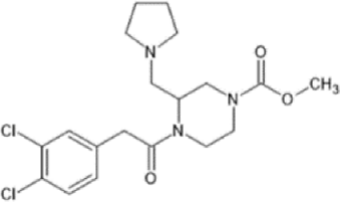	↑ Anti-nociception: spinal cord injury (0.32 μM, i.t., rat, M) and tail-flick (0.32 μM, i.t., rat, M) [18]	↓ Locomotor recovery in a contusion model [0.32 μM, rodent, M) 0.32 μM, i.t., rat, M] [18]	Aceves et al. (2016) [18]; Ko and Husbands (2009) [19]; Birch et al. (1991) [20]
	↑ Anti-nociception: tail-flick (0.01–0.1 g/kg, i.m., rhesus monkeys, M & F) [19]		
	↓ Itching: intrathecal morphine-induced scratching (0.1 μg/kg, i.m., rhesus monkeys, M & F) [19]		
	↑ Neuroprotection: global and cerebral ischaemia (3–30 μg/kg, s.c., gerbil, M & F) [20]		
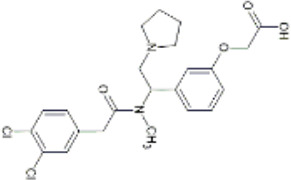 ICI-204,448	↓ Nociception: nerve-injured paw (40 µg/kg, i.pl., rats, M) [21]	↑ Hypothermia (5, 10 mg/kg s.c) in cold exposed rats (M) [22]	Barber et al. (1994) [3]; Peart et al. (2004) [9]; Shahbazian et al. (2002) [11]; Keïta et al. (1995) [21]; Rawls et al. (2005) [22]
	↓ Allodynia (40, 50 μg/kg, i.pl) in mechanical allodynia rat model, (M) [21]	n/e on sedation: rotarod test (100 mg/kg, i.v., mice) [3]	
	↓ Infarct size (0.3 mg/kg) in intact rat model (M) of myocardial infarction [8]		
	↓ Intestinal peristalsis (0.3–44.3 nM, guinea-pig small intestine) [11]		
ICI-199,441 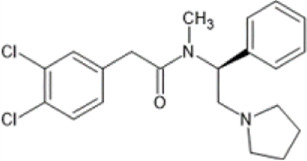	↓ Nociception: formalin footpad test (0.7–7 nM, i.pl., rat, M) [23]	ND	Obara et al. (2009) [23]; Endoh et al. (2000) [24]
	↓ Nociception: paw pressure test (0.54 mg/kg s.c., 0.074 mg/kg, i.m., rat, M) [24]		
	↓ Edema formalin induced inflammation (7 nM, i.pl., rat, M) [23]		
MB-1C-OH 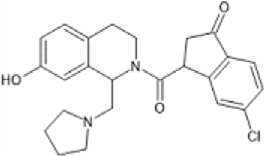	n.e. Anti-nociception: hot-plate and tail-flick (10 mg/kg, s.c., mice, M).	↓ Sedation (ED50 9.29 mg/kg s.c., mice, M)	Zhang et al. (2015) [25]
	↓ Nociception: acetic acid-induced writhing (EC_50_ 0.39 mg/kg, s.c., mice, M) [25]	↓ depression (ED_50_ 9.49 mg/kg s.c., mice, M) (≈3-fold) relative to U50,488 [25]	
Niravoline 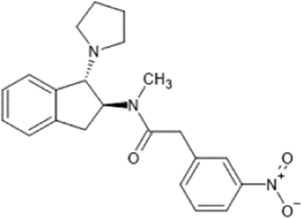	↓ Brain oedema: ischemia (1 mg/kg, i.v., rat, M) [26]	↑ Diuresis (1 and 3 mg/kg, i.v., rat, M) [27]	Ikeda et al. (1997) [26]; Moreau et al. (1996) [27]
Spiradoline (U62,066) 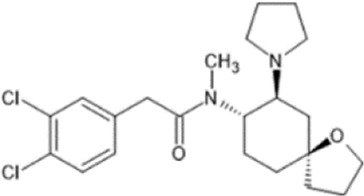	↑ Neuroprotection: (0.01–1 μM, rat, M) in hippocampal slices with hypoxia/hypoglycemia-induced glucose uptake deficit [28]	↑Diuresis (0.32 mg/kg, i.p., rats, M) [29]	Shibata et al. (1995) [28]; Yamada et al. (1989) [29]; Pugsley et al. (1998) [30]; Giardina et al. (1994) [31]
	↓ Heart rate and contractility isolated heart (100 μmol/kg, i.v., rat, M) [30]	↑Sedation (≥1.55 mg/kg, s.c., rat, M) rotarod test [31]	
	↓ Arrhythmia: Isolated rat heart (2–2.5 μmol/kg, i.v., M) [30]		
U50,488 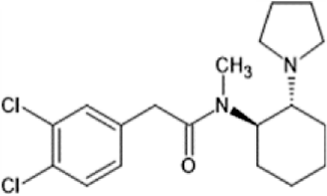	↓ Nociception: tail-flick, tail pinch, writhing tests (2.5 mg/kg, s.c., mice, M) [32]	↑ Aversion (10.0 mg/kg, i.p. rat, M) in cocaine induced place preference test [33]	Von Voigtlander et al. (1983) [32]; Suzuki et al. (1992) [33]; Kuzumaki et al. (2012) [34]; Ehrich et al. (2015) [35]; Kamei and Nagase (2001) [36]; Silvia et al. (1987) [37]; Privette and Terrian, (1995) [38]; Mei et al. (2016) [39]; Du et al. (2016) [40]; Paris et al. (2011) [41]; Song et al. (2021) [42]
	↓ Nociception in warm plate (4.4 mg/kg, s.c., M), hot plate (7.0 mg/kg, s.c., M), tail flick (32 mg/kg, s.c., M) and air writhing (1.4 mg/kg, s.c. rat, M)) [32]	↑ Sedation (10 mg/kg, i.p. mice, M) in CPP test [35]	
	↓ Tumor cell growth (15.6–250 μM, NSCLC cell lines) [34]	↑ Anxiolytic actions (10 and 1,000 μg/kg, i.p.) in elevated plus-maze test in rats (M) [38]	
	↓ Scratching (1–10 mg/kg, p.o., mice) [36]	↓ Novel object recognition in mice (1.0, 10.0 mg/kg, i.p. M) [41]	
	↓ Brain edema and neuronal injury (30 mg/kg, i.p.) in global cerebral ischemia in rat models (M) [37]		
	↑ Oligodendrocyte differentiation (0.5 µM) *in vitro* OPC cultures [39]		
	↑ Remyelination in the lysolecithin-induced demyelination mouse model (10 mg/kg/day, p.o., M & F) [39]		
	↑ Remyelination (1.6 mg/kg, i.p.) in EAE mice models (F) [40]		
	↑ Remyelination (1.6 mg/kg, i.p.) in cuprizone-induced demyelination mice model (F) [40]		
	↑ Learning and memory (1.25 mg/kg, s.c.) in Morris water maze test in mouse model (M) of Alzheimer’s disease [42]		
U69,593 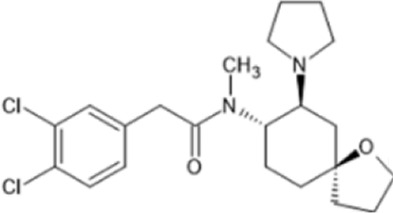	↓ Nociception: hot plate test (3.75 mg/kg, i.p., rat) [43]	↓ Respiration	Hughes et al. (1998) [14]; La Regina et al. (1988) [43]; Morani et al. (2009) [44]; France et al. (1994) [45]; Binder et al. (2001) [46]; Soulard et al. (1996) [47]; Valenza et al. (2017) [48]
	↓ Levodopa-induced dyskinesia in parkinsonism model (375 mg/kg, i.p., rat, M) [14]	↑ Motor activity, salivation (0.032–0.1 mg/kg, s.c., rhesus monkey) [45]	
	↓ Drug seeking: cocaine reinstatement test (0.3 mg/kg, s.c., rat, M) [44]	↑ Diuresis (0.3–3 mg/kg, s.c., rat, M) [47]	
	↓ Inflammation: Freund complete adjuvant (100 μg/paw, i.pl., rat, M) [46]	↑ CPA (0.32 mg/kg, s.c., rat, M) [48]	
Family: Benzomorphans			
Bremazocine 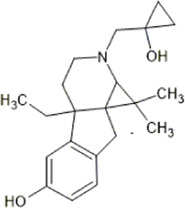	↓ Nociception: tail-flick (13.7 nM/mouse, i.c.v., mice, M) [49]	↓ Respiration rat (>8 mg/kg, s.c., mice)	Kuzmin et al. (2000) [7]; Horan et al. (1991) [49]; Nestby et al. (1999) [50]; Hayes and Tyers (1983) [51]
	↓ ethanol self- administration: ethanol self-administration test (0.1 mg/kg, s.c., rat, M) [50]	↑ Sedation (0.1–8 mg/kg, s.c., mice, M or F) [51]	
	↓ Motor activity: (0.312 mg/kg and higher, s.c., mice, M) [7]		
Family: Diphenethylamines			
HS665 (A), HS666 (B) 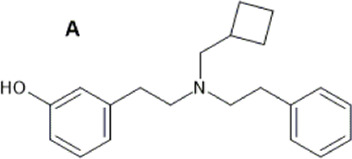 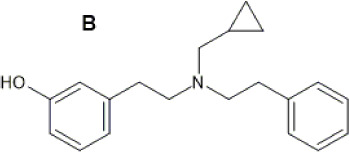	↓ Nociception: tail-flick (HS665 3.74 nM, HSS6 6.02 nM, i.c.v, mice, M) [52]	n/e motor performance in rotarod test [HS665 (10 nM) and HS666 (30 nM)].	Spetea et al. (2017) [52]
		n.e. CPA/CPP [HS665 (30 nM) and HS666 (150 nM., mice, M, i.c.v)] [52]	
Family Diazabicyclononanone			
HZ-2 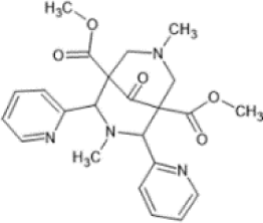	↓ Nociception: tail-flick (1.80–2.71 mg/kg, i.v., mice) [53]	↑ Sedation (4.27 mg/kg, i.p., mice)	Kögel et al. (1998) [53]
	↓ Nociception: formalin footpad (21.5 mg/kg, i.p., mice) [53]	↓ Motor coordination (≥14.7 mg/kg, i.p.)	
		n/e on respiratory rate (0.4–10 mg/kg, i.v., rat)	
		↓ Exploratory activity (4.27 mg/kg, i.p., mice) [53]	
Family: Ibogaine			
Noribogaine (O-desmethylibogaine/12-hydroxyibogamine) 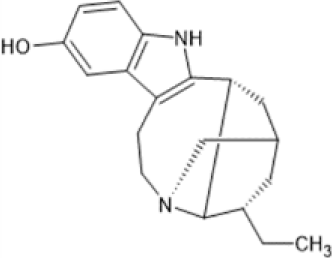	↓ Ethanol self-administration: two bottle choice (5–40 mg/kg, i.p., rat, M) [54]	ND	Rezvani et al. (1995) [54]; Chang et al. (2015) [55]
	↓ Nicotine self-administration: intravenous drug self-administration (25–50 mg/kg, p.o., rat, M) [55]		
Family: Morphinans			
Nalfurafine 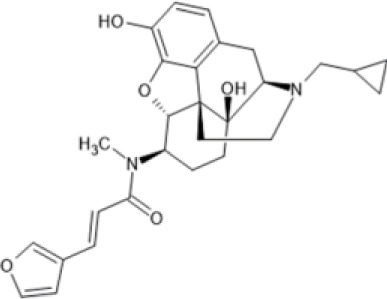	↓ Pruritis: substance P and histamine induced itch (100 μg/kg, p.o., mice, M) [56] and compound 48/80-induced itch (2.5–30 μg/kg, s.c., mice, M) [57]	n.e. CPA or CPP (5–20 μg/kg, s.c., M) [58, 24]	Endoh et al. (1999) [24]; Togashi et al. (2002) [56]; DiMattio (2016) [57]; Liu et al. (2019) [58]; Zhou and Kreek (2019) [59]; Denny et al. (2021) [60]; Ikeda et al. (2009) [61]; Inan et al. (2009) [62]
	↓ Nociception: tail-flick (ED_50_ 0.062 mg/kg, s.c., mice, M & F) [59]	n.e. anhedonia (sucrose preference test) (10 μg/kg, s.c.) and anxiety (elevated plus maze test) (10 μg/kg, s.c.) [24]	
	↓ Neuroinflammation: EAE model of MS (0.03–0.01 mg/kg, i.p., mice, F) [60]	n.e dysphoria.	
	↑ Remyelination: cuprizone model (0.01 mg/kg, i.p., mice, F) [60]	↑ Diuresis (5–10 μg/kg, s.c., rat, M) [62]	
	↓ Levodopa-induced dyskinesia, parkinsonism rat (10–30 μg/kg, s.c., rat, M) [61]		
SLL-039 and SLL-1206 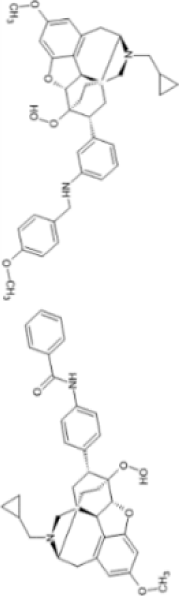	↓ Visceral nociception: acetic acid-induced writhing (1 mg/kg, i.p., mice, M)	n.e. sedation (0.5 mg/kg, i.p., mice); dysphoria (0.4–0.5 mg/kg, i.p., mice, M) [63]	Wei et al. (2021) [63]
	↓ Nociception: hot plate test SLL-039 (0.05, 0.1, 0.3 mg/kg; i.p., mice.) and SLL-1206 (0.06–1 mg/kg; i.p., mice, M).		
	↓ Scratching: acute pruritis induced by chloroquine SLL-039 (0.05–0.3 mg/kg; i.p, mice, M) and SLL-1206 (0.06–1 mg/kg; i.p, mice, M).		
	↓ Reward: morphine-induced CPP SLL-1206 (0.4–0.8 mg/kg, i.p., mice, M) [63]		
Family: Peptides			
CR665 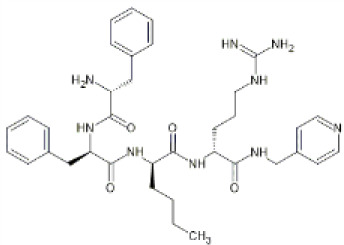	↓ Nociception: Freund adjuvant model (0.2–20 mg, s.c., rat, M) [47, 64]	ND	Binder et al. (2001) [46]; Soulard et al. (1996) [47]; Vanderah et al. (2008) [64]
	↓ Inflammation: Freund’s adjuvant model (30 and 100 μg/paw, i.pl., and 2 mg, s.c., rat, M) [46]		
Difelikefalin (CR845) 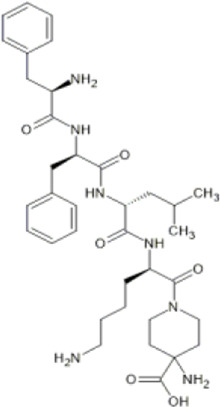	↓ Nociception: writhing test (0.09 mg/kg, i.v., mice, M & F) [65]	↓ Respiratory rate (at 25 min 2 mg/kg, i.v., mice, M & F) [65]	Wang et al. (2021) [65]
	↓ Allodynia: hind paw incision model and chronic constriction injury (3 mg/kg, i.v., rat, M) [65]		
	↓ Pruritis: 48/80-induced scratching (0.10 mg/kg, i.v., mice, M) [65]		
LOR17 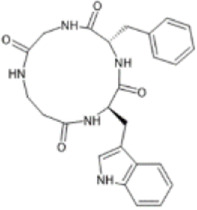	↓ Nociception: tail-flick (10.07 ± 0.36 mg/kg, i.p., mice, M)	n.e. motor coordination, locomotor, pro-depressant-like behaviours (10 mg/kg, s.c., mice, M) [66]	Bedini et al. (2020) [66]
	↓ Nociception: writhing test (5.74 ± 0.46 mg/kg, i.p., mice, M)		
	↓ Thermal hypersensitivity: oxaliplatin-induced neuropathic nociception (10–20 mg/kg, s.c., mice, M) [66]		
Family: Phenothiazines			
Apadoline 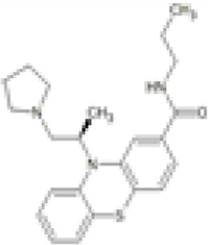	↓ Nociception: writhing models (0.08 mg/kg, s.c., mice and rat) [67]	n/e aggression (1–4.5 mg/kg, i.v.) [68]	Fardin et al. (1990) [67]; Stutzmann et al. (1995) [68]
	↓ ECG and ECoG (1–4.5 mg/kg, i.v., Baboons) [68]		
Family: Terpenoids			
β-THP SalB 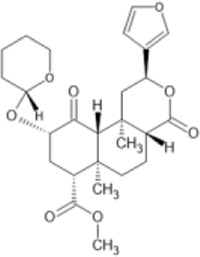	↑ Duration: tail-flick (2 mg/kg dose, s.c., mice (M), compare to SalA) [69, 70]	n/e Sedation, depression, FST, anxiety, locomotor activity (1 and 2 mg/kg, i.p., rat, M) [71]	Simonson et al. (2015) [69]; Paton et al. (2017) [70]; Ewald et al. (2017) [71]
	↓ Allodynia: paclitaxel-induced neuropathy (EC_50_ 1.4 mg/kg, s.c., mice, M) [70]		
	↓ Nociception: Intradermal formalin (1–2 mg/kg, i.p., mice, M) [70]		
	↓ Inflammation (2 mg/kg, i.p., mice, M) [69]		
	↓ Cocaine induced hyperactivity (1 mg/kg, i.p., rat, M) [71]		
Collybolide 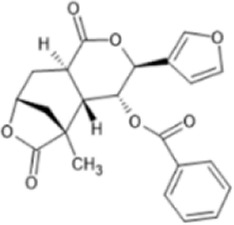	↓ Nociception: tail-flick assay (2 mg/kg, i.p., mice, M).	↑ Aversion in the CPA test (2 mg/kg, i.p., mice, M) [72]	Gupta et al. (2016) [72]
	↓ Depression: FST (2 mg/kg, i.p., mice, M)		
	↑ Anxiogenic activity: open field test (2 mg/kg, i.p., mice, M)		
	↓ Pruritus: chloroquine-mediated itch (2 mg/kg, i.p., mice, M) [72]		
EOM SalB 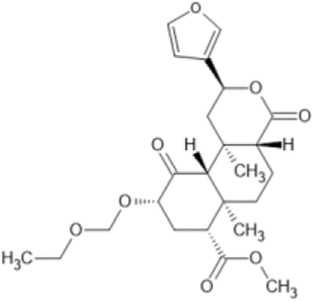	↑ Metabolic stability: rat (M) liver microsome (10 μM) [71]	n.e. anxiety, depressive-like effects (0.1–0.3 mg/kg, i.p, rat). n/e on locomotor activity, open arm times or swimming behaviours (0.1 and 0.3 mg/kg, i.p., rat, M) [71]	Ewald et al. (2017) [71]; Kaski et al. (2019) [73]; Paton et al. (2021) [74]
	↓ Cocaine-seeking behaviour and hyperactivity, self-administration (0.1–0.3 mg/kg, i.p., rat, M) [71]		
	↑ Spinal anti-nociception (1 mg/kg, i.p., mice, M &F) [73]		
	↓ EAE disease severity, EAE (0.1–0.3 mg/kg, i.p., mice, F) [74]		
	↓ Immune cell infiltration		
	↑ Myelin levels in the spinal cord in EAE (0.1–0.3 mg/kg, i.p., mice, F)		
	↑Number of mature oligodendrocytes		
	↑ Number of myelinated axons		
	↑ Myelin thickness: cuprizone-induced demyelination (0.1–0.3 mg/kg, Mice, i.p., F) [74]		
Mesyl SalB 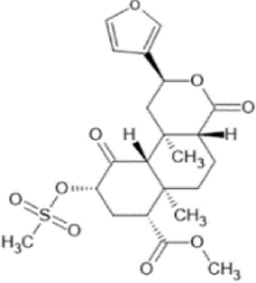	↓ Nociception: tail-flick (1 mg/kg, i.p., mice, M) [69]	n.e. aversion, anxiety, or learning and memory (1–2 mg/kg, i.p., rat, M) [75]	Simonson et al. (2015) [69]; Kivell et al. (2018) [75]
	↑ duration of action (Vs. SalA,1 mg/kg, i.p., mice, M) [69]	n.e. Sedation (1–2 mg/kg, i.p., rat, M) [75]	
	↓ Cocaine induced hypersensitivity (0.3 mg/kg, i.p., rat, M) [75]		
	n.e. Sucrose intake (0.3–1 mg/kg, i.p., rat, M) [75]		
	n.e Memory impairment: novel object recognition (0.3–1 mg/kg, i.p., rat, M) [75]		
Salvinorin A 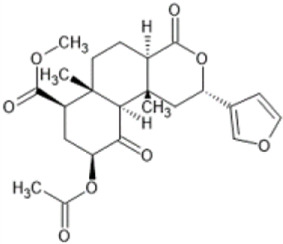	↓ Nociception: tail-flick (1–4 mg/kg, s.c., mice, M) [70, 76]	↑ CPA,	Paton et al. (2017) [70]; McCurdy et al. (2006) [76]; Zhang et al. (2005) [77]; Dong et al. (2018) [78]; Butelman et al. (2009) [79]; Braida et al. (2011) [80]
	↑ Function; forebrain ischemia (10 μg/kg, i.v., rat) [78]	↓ Locomotion (1–3.2 mg/kg, i.p., mice, M) [77]	
	↓ Inflammation: intradermal formalin (2 mg/kg, i.p., mice) [70]	↓ Sedation (0.01–0.032 mg/kg, i.v., monkeys (M & F) [79]	
		↓ Learning and memory (80–640 μg/kg s.c., rat, M) [80]	
		↑ Anxiogenic effects (0.3 mg/kg, i.p., rat, M) [80]	
MOM SalB 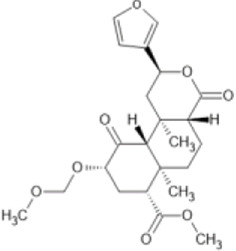	↓ Nociception: hot plate (0.5–5 mg/kg, i.p., rat, M)	↑ Hypothermia (0.5–5 mg/kg, i.p. rat, M)	Wang et al. (2008) [81]
	↑ Hypothermia: (0.5–5 mg/kg, i.p., rat, M)	↑ Sedation (0.01–1 mg/kg, i.p., mice, M) [81]	
	↓ Motor activity: home cage activity (0.05–1 mg/kg s.c., mice, M) [81]		
Family: Triazole			
Triazole 1.1 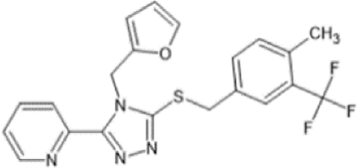	↓ Nociception (30 mg/kg, i.p., mouse, M) Tail flick assay [82]	n/e sedation, open field test	Zhou et al. (2013) [82]; Brust et al. (2016) [83]
	↓ Pruritis (1–15 mg/kg, s.c., mice, M) in chloroquine phosphate–induced scratching responses [83]	n.e. dysphoria (3–30 mg/kg, i.p., rat) [83]	
	↑ Anti-nociceptive effects: ICSS abdominal nociception model (24 mg/kg, i.p., rat) [83]		

Abbreviations: CPP, conditioned place preference; CPA, conditioned place aversion; EAE, experimental autoimmune encephalomyelitis; ECG, electrocardiogram; ECoG, electrocorticogram; ED_50_, median effective dose; F, female; FST, force swim test; ICSS, intracranial self-stimulation; i.c.v., intracerebroventricular; ICSS, intercranial self-stimulation; ID_50_, inhibitory dose; i.m., intramuscular; i.p., intraperitoneal; i.pl., intraplantar; i.t., intrathecal; i.v., intravenous; M, male; ND, not determined; n.e., no effect; p.o., per oral; s.c., subcutaneous.

**TABLE 2:** Therapeutic effects and side effects of KOR agonists in clinical studies.

KOR agonist	Clinical therapeutic effects	Side effects	References
ADL 10-0101	Phase I: completed: safety assessment in healthy volunteers (Adolor Corporation) (10 μg/kg/min, i.v. s.e all) [1]	↑Headache, restlessness n/e BP, HR, respiratory rate, nausea (10 μg/kg/min, i.v.) [1]	Eisenach et al. (2003) [1]
	Phase II: Effectiveness in reducing pain in patients suffering from chronic pancreatitis (10 μg/kg/min, i.v.) *n* = 6; s.e., all; age 18–60 years [1]		
Asimadoline	Phase II: completed (12/2007). Safety and efficacy of asimadoline (0.15, 0.5, 1.0 mg, p.o.) in the treatment of patients with IBS *n* = 596; s.e., all; age 18–79 years [NCT00454688].	ND	
	Phase II: completed (3/2007). Effect of Asimadoline (0.5–1 mg, p.o) on acute pain attacks (IBS) *n* = 100; s.e., all; age 18–65 years [NCT00955994].		
	Phase II: completed (6/2017). Safety, tolerability, and efficacy of asimadoline (5 mg, p.o.) in patients with pruritus associated with atopic dermatitis *n* = 249; s.e., all; age 18 years and above [NCT02475447].		
	Phase III: completed (6/2013). Safety and efficacy of asimadoline (0.5 mg, p.o.) in treating Diarrhea-Predominant IBD *n* = 611; s.e., all; age 18–79 years [NCT01100684].		
	Phase II: terminated for post-operative ileus *n* = 35; s.e., all; age 18–80 years (poor enrolment) [NCT00443040].		
Enadoline	Pilot Study: Characterisation; pharmacodynamic effects (10–80 80 µg/70 kg, i.m.) *n* = 9; s.e., all; age 22–47 years	Psychotomimetic effects, skin prickling, unsteady gait (160-µg/70 kg, i.m.) [2]	Walsh et al. (2001) [2]
Fedotozine	Phase II: completed (1994):	No pattern of drug related adverse effects [3]	Fraitag et al. (1994) [3]; Read et al. (1997) [4]; Barber and Gottschlich (1997) [5]; Delvaux (2001) [6]
	↓ Post-prandial fullness, bloating, nausea		
	↓ Abdominal pain (30 and 70 mg, p.o.) *n* = 146; s.e., all; age 43–55 years [3]		
	Phase III: completed (1997): effective for the relief of functional dyspepsia (30 mg thrice daily, p.o.) *n* = 333; s.e., all [4]		
	Phase III: terminated: IBS and dyspepsia; terminated (lack of efficacy) [5, 6]		
Niravoline	Phase II: completed: Effective treatment of patients with cirrhosis and water retention (0.1–2 mg, i.v.) *n* = 18; s.e., all; age 52± years [7]	↑ Diuresis (0.1–2 mg, i.v.)	Gadano et al. (2000) [7]
		↑ Aquaretic effect (0.5 and 1 mg, i.v.) [7]	
Spiradoline (U62,066)	Phase I: completed (12/2009): Bipolar Depression: *n* = 24; s.e., male; age 21–55 years [NCT00988949].	↑ Diuresis (1.6 and 4.0 μg/kg, i.m.)	Peters et al. (1987) [8]; Chappell et al. (1993) [9]; Ur et al. (1997) [10]
	Phase I: Diuretic actions (2–6 μg/kg, i.m.)[8]	↑ Prolactin, growth hormone and cortisol levels (1.6 and 4.0 μg/kg, i.m.) [10]	
	Clinical study: Effect of Spiradoline on Tourette’s syndrome.		
	↓ Frequency of tics, Low doses (0.8 μg/kg, i.m) higher doses either worsened tics, or had n.e. *n* = 10; s.e., male; age 20–47 years [9]		
Noribogaine (O-desmethylibogaine/12-hydroxyibogamine)	Phase I: completed (2014): Safety, tolerability, pharmacokinetic, and pharmacodynamic profiles of noribogaine (3–60 mg/kg, p.o.) *n* = 36; s.e., male; age 18–55 years [ACTRN12612000821897] [11]	Headache (3 mg), Epistaxis (placebo) [11]	Glue et al. (2015) [11]
	Phase I and II: recruiting patients (Exp. completion date: 9/2023) Efficacy, safety and tolerability of Ibogaine for opioid withdrawal patients (3–12 mg/kg, p.o.) *n* = 110; s.e., all; age 18–55 years [NCT05029401]		
Nalfurafine	Clinically approved (Japan) (2009) Remitch for medication-resistant pruritus in patients with hemodialysis [12]	Insomnia (≥3% (5 μg/kg, p.o.) [13]	Inan et al. (2009) [12]; Kumagai et al. (2010) [13]
	Phase III: completed (9/2009): For the treatment of pruritus in patients receiving hemodialysis (5 μg, p.o.) *n* = 104; s.e., all; age 20 years and older [NCT01513161].		
	Phase II: completed (3/2018): Nalfurafine as a treatment for pruritus in patients with primary biliary cholangitis *n* = 44; s.e., all; age 18 years and older [NCT02659696].		
	Phase II: completed (12/2009): Pruritus in patients with chronic liver disease (2.5–10 μg, p.o.) *n* = 120; s.e., all; age 20 years and older [NCT00638495].		
	Phase III: recruiting patients (Exp. completion date 10/2021): Efficacy, safety and plasma concentration of nalfurafine for treatment of refractory pruritus (5 μg, p.o.) *n* = 133; s.e., all; age 18 years and older [NCT04728984].		
CR665	Phase II: completed (2009):	No serious side effects Paresthaesia, dizziness, somnolence and increased prolactin levels [14]	Arendt-Nielsen et al. (2009) [14]
	↓ Visceral pain in a human model of oesophageal distension (0.36 mg/kg; i.v.) *n* = 18; s.e., male; age 19–43 years [14]		
Difelikefalin (CR845)	FDA approved (8/2021): (Dose: 0.5 μg/kg, 3x weekly, i.v.) *n* = 222; s.e., all; age 18–25 years [NCT03998163] [15]	Common adverse reactions: diarrhoea, dizziness, nausea, gait, hyperkalaemia. headache 0.5 μg/kg 3x weekly, i.v.) occurring in ≥ 2% of recipients (*n* = 424) [16]	Wang et al. (2021) [15]; Deeks (2021) [16]
	Phase II: completed (1/2016): osteoarthritis of the hip or the knee (0.25–5 mg, p.o.) n = 81; s.e., all; age 25 and above [NCT02524197]		
	Phase II: completed (4/2021): Pruritus (atopic dermatitis) (0.25–1 mg, p.o.) *n* = 401; s.e., all; age 18–80 years [NCT04018027]		
	Phase II: recruiting patients (Est. completion date, 3/2022): Pruritus (notalgia paraesthetica) (2 mg, p.o.) *n* = 120; s.e., all; age 18–80 years [NCT04706975]		
	Phase II: recruiting patients (Est. completion date, 6/2022): Pruritus (primary biliary cholangitis) (1 mg, p.o.) *n* = 60; s.e., all; age 18–80 years [NCT03995212]		
Apadoline	Phase I: Reduced pain (0.1, 0.5, and 1.0 mg, p.o.) *n* = 20; s.e., male [17]	Mild drowsiness and headache (1 mg, p.o.) [17]	Lötsch et al. (1997) [17]
Salvinorin A	Drug Effect: Phase I and II: completed (3/2020). Effects on human brain activity and connectivity, *n* = 13; age 21–50 years; s.e., all [NCT03418714]	↑ Psychoactive effects inh. (0.25 mg, inh.)	Maqueda et al. (2015) [18]; Addy (2012) [19]; MacLean et al. (2013) [20]
	Phase I: completed (11/2013): Hallucinogenic effects n = 14; age 21–65 years; s.e., all [NCT00996411].	↑ Synesthesia (inh.0.25 mg) [18]	
	Phase I: ongoing (Est. completion date: 12/2021): Psychotomimetic Effects in healthy people *n* = 66; age 18–45 years; s.e., all [NCT00700596].	↑ Hallucinations (inh.100 µg) [19]	
	Phase I: completed (2019): For effects on mood and performance completed *n* = 20; age 21–50 years; s.e., all [NCT02033707].	↑ Dissociative effects (inh. 0.375–21 μg/kg) [20]	

Abbreviations: BP, blood pressure; HR, heart rate; i.m., intramuscular; i.v., intravenous; inh, inhalation; ND, not determined; n.e., no effect; n, number of participants; p.o., per oral; s.c., subcutaneous; s.e., sexes eligible for study.

## 3 Diseases for Which the Kappa-Opioid Receptors Has Therapeutic Potential

### 3.1 Pain

KORs are currently being investigated as a therapeutic target for the treatment of pain because KOR activation has potent anti-nociceptive effects in various models of acute (mechanical, thermal and chemical) (Briggs et al., 1998; Beck et al., 2019; Escudero Lara et al., 2021), inflammatory (Paton et al., 2017), neuropathic (Sounvoravong et al., 2004), and cancer pain (Edwards et al., 2018). The use of KOR agonists for the treatment of pain are therapeutically desirable due to the presence of anti-nociceptive effects without abuse potential (Beck and Dix, 2019) or respiratory depressive effects (Viscusi et al., 2021; Wang et al., 2021).

However, the clinical use of traditional KOR agonists has previously been limited due to their adverse effects identified in preclinical studies, such as sedation (Zhang et al., 2015), conditioned place aversion (Ehrich et al., 2015), and pro-depressive effects (Carlezon et al., 2006). The traditional KOR agonists 2-(3,4-dichlorophenyl)-*N*-methyl-*N*-[(1*R*,2*R*)-2-pyrrolidin-1-ylcyclohexyl]acetamide (U50,488) and *N*-methyl-2-phenyl-*N*-[5*R*,7*S*,8*S*]-7-pyrrolidin-1-yl-1-oxaspiro [4,5]decan-8-yl]acetamide (U69, 593) are potent and selective full agonists at KOR (Von Voigtlander and Lewis, 1982; Von Voigtlander et al., 1983; Emmerson et al., 1994). These traditional KOR agonists have since served as structural scaffolds for the development and synthesis of novel KOR agonists with the aim of developing KOR agonists devoid of side effects. Many novel KOR agonists have also been either isolated or synthesised and used as novel structural scaffolds.

Salvinorin A (SalA), isolated from the plant *Salvia divinorum* is a potent, centrally acting KOR agonist that produces anti-nociceptive effects in a dose-dependent manner in animal models of visceral, thermal (McCurdy et al., 2006), inflammatory (Aviello et al., 2011), and neuropathic pain (Coffeen et al., 2018). However, its clinical progression has been limited due to its short duration of action (McCurdy et al., 2006), potent hallucinogenic effects (Prisinzano, 2005), in addition to inducing aversion (CPA) (Zhang et al., 2015), anxiety (elevated plus maze) (Ewald et al., 2017), sedation (facial relaxation and ptosis) (Butelman et al., 2019) and motor incoordination (inverted screen test) (Fantegrossi et al., 2005) reviewed in (Paton et al., 2020a). However, modifications to the structure of SalA have yielded compounds with increased anti-nociceptive potency. For example, β-tetrahydropyran salvinorin B (β-THP SalB) has been shown to display potent anti-nociceptive effects in rodent models of thermal, inflammatory, and neuropathic pain. The dose–response effects of β-THP SalB, SalA and U50,488 revealed differences in both potency (ED_50_) with β-THP SalB > SalA > U50,488 and efficacy (Emax) in the hot water tail-withdrawal assay. In a chemotherapy-induced neuropathic pain model, both SalA and β-THP SalB dose-dependently reduced mechanical and cold allodynia to pre-paclitaxel levels, with the novel SalA analogue, β-THP SalB demonstrating increased potency over the parent compound (Paton et al., 2017). Furthermore, additional analogues of SalA, 16-Bromo SalA and 16-Ethynyl SalA proved to be more potent in attenuating nociception in comparison to U50,488 and had a longer duration of action in the warm water tail-flick assay in mice. These novel compounds were also effective in reducing nociceptive behaviours in the intraplantar formaldehyde model of inflammatory nociception with neither compound displaying anxiolytic effects (Paton et al., 2020b).

Another KOR agonist, collybolide, isolated from the fungus *Collybia maculate*, was found to be highly selective and G-protein biased. Collybolide, displayed anti-nociceptive effects in the tail-flick assay, similar to SalA, and reduced scratching behaviours in a mouse model of pruritis (Gupta et al., 2016). Collybolide also displayed fewer side effects with no pro-depressive behaviours observed in the force swim test (mice spent less time immobile) or anxiolytic effects in the elevated zero maze (mice spent more time in the open arms), or reduced locomotor activity in open-field tests (no effect on distance travelled). However, collybolide did exhibit aversive behaviour in the condition placed aversion test in mice (Gupta et al., 2016).

Triazole 1.1 is a selective KOR agonist that displays significant G-protein signalling bias (Zhou et al., 2013; Lovell et al., 2015). This compound was identified through a high-throughput screening assay which identified selective chemotypes that had agonistic properties at KOR (Frankowski et al., 2012). Triazole 1.1 produces anti-nociceptive effects in the tail-flick assay comparable to that of U50,488. In addition, it was also found to be efficacious in suppressing scratching in a mouse model of non-histamine induced pruritis. Triazole 1.1 showed an improved safety profile with no change in locomotor activity in the open field assay in mice, or dysphoria observed in intracranial self-stimulation assays in rats in comparison to U50,488. In addition, triazole 1.1 did not induce decreases in dopamine levels in comparison to U50,488 which decreased dopamine levels within the nucleus accumbens in a dose-dependent manner (Brust et al., 2016). In rhesus monkeys, triazole 1.1 (0.01–0.32 mg/kg) did not induce sedative or motor impairment effects (Huskinson et al., 2020). Furthermore, in rats, triazole 1.1 reduced oxycodone self-administration, and co-administration of oxycodone and triazole 1.1 enhanced anti-nociceptive effects (Zamarripa et al., 2021). Thus, trizole 1.1 demonstrates an improved therapeutic index in comparison to balanced KOR agonists such as U50,488. Together, these data support that G-protein biased KOR agonists have reduced side effects.

More recently, peripherally restricted KOR agonists such as those advanced by Cara therapeutics have been developed in attempts to reduce side-effects. CR845 (also known as difelikefalin) and CR665 (also known as FE-200665) both exhibit potent anti-nociceptive effects in preclinical models of inflammatory, visceral (Binder et al., 2001; Arendt-Nielsen et al., 2009) and neuropathic pain without gastrointestinal side effects (Gardell et al., 2008). CR845 completed phase III clinical trials (Beck and Dix, 2019) and recently received FDA approval in the United States under the name Korsuva for the treatment of pruritis in adults with chronic kidney disease who are undergoing haemodialysis (Deeks, 2021). In a single-dose crossover study involving healthy volunteers, intravenous (i.v.) CR845 at a dose of 1.0 or 5.0 μg/kg was not associated with respiratory depression (Viscusi et al., 2021). A detailed review of CR845’s performance in pre-clinical and clinical evaluations can be found in Lipman and Yosipovitch (2021).

Structural derivatives of CR665 have also been developed in attempts to increase bioavailability. JT Pharmaceuticals developed JT09, which was shown to reduce acetic acid–induced writhing behaviours in rats following oral administration (30 mg/kg) (Hughes et al., 2013). JT09 showed limited centrally mediated side effects and did not induce rewarding (cocaine self-administration test), pro-depressive (no increase in inactivity in the forced swim test), or sedative effects (no change in open field activity) in preclinical rat models (Beck et al., 2019).

Nalfurafine (also known as TRK-820; marketed clinically as Remitch) is the only full, KOR-selective, centrally-acting KOR agonist approved for clinical use for the treatment of pruritis in Japan and South Korea (Nakao and Mochizuki, 2009). In this patient group, nalfurafine has proven to be safe and well tolerated (Kozono et al., 2018). Preclinical studies with nalfurafine have shown anti-nociceptive effects that have greater potency than U50,488 and a longer duration of action than the MOR agonist, morphine. There is also evidence that KOR agonists potentiate the effects of MOR agonists. TRK-820 10 and 30 μg/kg/subcutaneously (s.c.) co-administered with morphine enhanced the antinociceptive effects in the mouse hot plate test (Endoh et al., 1999). However, nalfurafine is not suitable as a clinical analgesic as it has side effects at doses that induce analgesia (Inan et al., 2009; Kaski et al., 2019).

Together, these pre-clinical and clinical studies demonstrate that KOR agonists have the potential to be developed into safe pharmacotherapies without abuse liability and may be useful in addressing the opioid epidemic.

### 3.2 Pruritis

Pruritis is a sensation that induces itching or scratching, leading to irritated skin and is one of the most common reasons people seek dermatologist advice. It is well established that activation of KOR induces anti-pruritic effects due to being involved in the modulation of the sensation of itch. In contrast, the use of KOR antagonists, nor-BNI and 5′GNTI, promote a scratching response in wild-type mice when injected subcutaneously (s.c.) and the effect of these antagonists was less severe in KOR knock-out mice (Morgenweck et al., 2015). In contrast, MOR agonists are shown to promote itch, while MOR antagonists have the ability to supress itch in wildtype mice (Ko, 2015).

In 1984, Gmerek and Cowan identified that the systemic administration of the early benzomorphan family of KOR agonists significantly decreased scratching in rats in a dose-dependent manner in a bombesin-induced model of itch (Gmerek and Cowan, 1984). Later, they also showed that U50,488 and tifluadom were also effective in attenuating scratching in the same rat model (Cowan and Gmerek, 1986). Furthermore, the peripherally restricted KOR agonist ICI 204,448 was able to inhibit scratching behaviours in the chloroquine-induced model of itch in mice (Inan and Cowan, 2004). Nalfurafine is more potent than U50,488 in preclinical tail-flick and acetic acid induced mouse models of nociception (Nagase et al., 1998). Nalfurafine has also been found to inhibit pruritus induced by compound 48/80 which stimulates histamine release and induces mast cell degeneration (Wang et al., 2005; Inan et al., 2009; Schemann et al., 2012), substance P (a peptide that induces itching) (Hägermark et al., 1978; Togashi et al., 2002; Umeuchi et al., 2003), histamine (Togashi et al., 2002), and chloroquine phosphate (Inan and Cowan, 2004). These studies have identified that nalfurafine has high potency in these models of itch without developing tolerance. In pre-clinical studies, CR845 (difelikefalin, brand name Korsuva) has proved to be highly effective in attenuating scratching behaviours induced by two models of itch, compound 48/80 and the KOR antagonist 5′GNTI, in a dose-dependant manner (Cowan et al., 2015). Another peripherally restricted KOR agonist, HSK21542, can also attenuate compound 48/80-induced itch in mice with an inhibitory rate of 99.8% in comparison to nalfurafine which reduced the scratching responses at an inhibitory rate of 94%. HSK21542 was also shown to have a higher potency than the recent FDA approved drug, Korsuva (Wang et al., 2021).

Although nalfurafine is widely use in Japan, the European Medicines Agency (EMA, 2013) denied its clinical approval for use in Europe for the same indications due to non-significant results regarding effectiveness in comparison to placebo controls. In contrast to its oral delivery approved for use in Japan, this EMA trial utilised intravenous administration, and this different route of administration may explain differences in efficacy and side-effects. In a phase III clinical trial involving 378 patients, administration of CR845 (Korsuva) significantly reduced itching and improved patients’ quality of life. The peripheral actions of this drug reduced the occurrence of dysphoria and hallucinations at therapeutic doses (Fishbane et al., 2020). These pre-clinical and clinical studies signal that KOR agonists play an important role in pruritis and inflammation and may be developed into effective pharmacotherapies.

### 3.3 Multiple Sclerosis

MS is an autoimmune disease of the central nervous system (CNS) (Karussis, 2014), affecting 2.8 million individuals worldwide (Walton et al., 2020). The disease pathology consists of demyelination in the grey and white matter of the CNS, and the symptoms vary depending on the severity and location of lesions. Common symptoms are limb and facial weakness, optic neuritis, cognitive dysfunction, fatigue, pain, and bladder dysfunction (Muto et al., 2015; Rommer et al., 2019).

The existing treatment options for relapsing-remitting MS include immunosuppressant and immunomodulatory agents and immune reconstitution therapies that can attenuate immune-mediated damage in the context of MS (Dobson and Giovannoni, 2019; Hauser and Cree, 2020). However, given the current inability of clinicians to predict and prevent the onset of MS and the lack of highly effective treatments for progressive MS (Hauser and Cree, 2020; Krajnc et al., 2021), there is an ongoing unmet need for remyelination therapies that repair the damaged myelin (Bove and Green, 2017).

Recent studies have shown that activation of KOR may be an effective strategy for promoting remyelination and functional recovery in preclinical models of MS. Several key papers supporting the therapeutic potential of KOR agonists as remyelinating agents have recently been published (Du et al., 2016; Mei et al., 2016; Thell et al., 2016; Tangherlini et al., 2019; Tangherlini et al., 2020; Denny et al., 2021; Paton et al., 2021).

Du et al. (2016) showed that genetic deletion of KOR exacerbated the symptoms resulting from experimental autoimmune encephalomyelitis (EAE) in mice, and that KOR agonists U50, 488 and asimadoline alleviated the symptoms of EAE in wild-type mice. The disease-modifying effects of U50, 488 were absent in KOR-knockout mice, providing strong evidence that the effects were KOR mediated. Immunolabeling for myelin and oligodendrocyte progenitor cells (OPCs) revealed that U50, 488 promoted OPC differentiation into mature myelinating oligodendrocytes (OLs) and enhanced remyelination in EAE. In a cuprizone-toxin-induced model of demyelination, mice administered with U50, 488 also showed enhanced levels of remyelination compared to vehicle treated mice (Du et al., 2016).

In a study by Mei et al. (2016), a library of ∼250 compounds targeting G-protein coupled receptors (GPCRs) was screened for their ability to promote differentiation of mouse and rat OPCs into mature OL, and they found that a cluster of 10 KOR agonists (U50,488, ICI-199441, U54,494, matrine, N-MPPP, BRL52537, GR89696, dynorphin B, 6′-GNTI, and SalA) successfully promoted OPC differentiation *in vitro*. U50,488 was the most effective. They also found that KOR antagonism inhibited OPC differentiation. The KORs were found to be expressed on the processes and somata of OPCs. Mei et al. (2016) also observed that administration of U50,488 accelerated OPC differentiation into mature OLs and promoted axon remyelination in lysolecithin-induced focal lesions in wild-type mice. Experiments from KOR-knockout mice provided evidence that effects were KOR dependent. In addition, when KOR was selectively deleted on OPCs, U50,488 failed to differentiate OPCs into mature OL (Mei et al., 2016).

Additional studies have provided further evidence that KOR agonists promote remyelination and recovery in mouse models of MS. Tangherlini et al. (2019) synthesised a series of quinoxaline-based KOR agonists and showed that two of these KOR agonists ameliorated EAE paralysis in mice. Tangherlini et al. (2020) also observed that treatment with KOR agonists reduced immune cell infiltration into the CNS. They also confirmed that immunomodulatory effects of these compounds depended on the presence of KOR (Tangherlini et al., 2020). Oral administration of a plant-derived peptide called [T20K]kalata B1 also reduced demyelination and improved functional EAE behavioural scores (level of paralysis) in mice. [T20K]kB1 also exerted long-lasting and protective T-cell antiproliferative properties and reduced the levels of inflammatory cytokines including IL-2, IFN-γ, and IL-17A(Thell et al., 2016). A recent publication confirmed that [T20K]kalata B1 is a KOR agonist (Muratspahić et al., 2021).

Recently, Denny et al. (2021) reported that the selective KOR agonist, nalfurafine, promoted functional recovery and remyelination and reduced CNS immune-cell-infiltration in EAE in a KOR dependent manner in mice. Nalfurafine also promoted remyelination in the cuprizone model of demyelination, providing evidence that KOR agonism promotes remyelination in the absence of peripheral immune cell infiltration (Denny et al., 2021). Furthermore, they showed that nalfurafine reduced immune cell infiltration into the CNS and reduced the expression of the proinflammatory cytokine interferon gamma in both CD4+ and CD8+ T cells. Nalfurafine also promoted a more immunoregulatory environment by decreasing responses from pro-inflammatory Th17 cells. These findings are important as nalfurafine is a drug with existing clinical usage and is safe and well-tolerated clinically, although insomnia was identified as common side effect (Kumagai et al., 2010). Preclinical research has shown that nalfurafine (5–30 μg/kg) does not cause CPA, anhedonia, sedation, or motor incoordination associated with traditional KOR agonists (Liu et al., 2019) at doses that promote remyelination in MS models (10 μg/kg) (Denny et al., 2021).

Paton et al. (2021) recently showed that a SalA analogue called ethoxymethyl ether salvinorin B (EOM SalB) promoted remyelination in EAE and cuprizone-induced demyelination models in mice. In EAE, EOM SalB decreased disease severity (paralysis), decreased CNS immune-cell-infiltration, and increased myelin levels. In the cuprizone-induced demyelination model, EOM SalB increased the number of mature oligodendrocytes, the number of myelinated axons, and the thickness of myelin within the corpus callosum. Furthermore, EOM SalB is a G-protein biased KOR agonist and has been shown to have reduced side-effects in preclinical studies (Ewald et al., 2017).

These findings are ground-breaking, as remyelination therapies for MS are highly sought-after. The MS medications market exceeds US$25 billion per year, driving the development of novel pharmacotherapies targeting repair and recovery in MS, particularly for progressive forms of the disease where there are no successful treatment options. While MS is the most common demyelinating disease, there are many other diseases in which myelin is damaged or dysregulated. Therefore, there is potential that KOR agonists may promote remyelination in other such conditions, including neuromyelitis optica (Argyriou and Makris, 2008), acute disseminated encephalomyelitis (Young et al., 2010), Skogholt disease (Aspli et al., 2015), adrenoleukodystrophy (Aubourg, 2015), and other leukodystrophies (van der Knaap and Bugiani, 2017). Interestingly, optic neuritis is among the most common initial manifestations in relapsing remitting MS patients (Hojjati et al., 2015; Kale, 2016). However, it remains to be determined whether KOR agonism promotes remyelination in other demyelinating diseases.

### 3.4 Alzheimer’s Disease and Cognitive Dysfunction

AD is a neurodegenerative disease pathologically characterised by extracellular β-amyloid deposits and the accumulation of hyperphosphorylated tau (Long and Holtzman, 2019). AD is a leading cause of dementia (Arvanitakis et al., 2019), but only symptomatic treatments are currently available (Yiannopoulou and Papageorgiou, 2020). Given that AD is a major and growing burden on healthcare systems globally (Wong, 2020), developing effective treatments is a key focus area for drug development.

Although demyelination is not the major pathology of AD, demyelination occurs in AD (Bouhrara et al., 2018) and there is growing evidence to suggest that KOR agonism may have a protective role in AD. Hypermethylation within the promoter region of *OPRK1* (study based on human peripheral blood samples), the gene which encodes KOR, is associated with an increased risk of AD (Ji et al., 2015). Increased KOR binding was found in the dorsal and ventral putamen and in the cerebellar cortex in coronal sections of postmortem brains from AD patients (Mathieu-kia et al., 2001). Also, elevated dynorphin levels were reported in postmortem samples of AD patients (Ménard et al., 2014). A recent study by Song et al. (2021) reported that U50,488, when administered in a mouse model of AD, promoted enhanced learning and memory in the Morris water maze test. In this study, (APP)/presenilin-1 (PS1) mice treated with U50,488 (1.25 mg/kg) showed a significant improvement in their cognitive abilities. In U50,488 treated APP/PS1 mice, amyloid-beta (Aβ) plaque deposition was decreased in the prefrontal cortex and hippocampus. Within this AD model there was reduced damage to hippocampal neurons, reduced microglia-induced pyroptosis, and improved synaptic plasticity which was partially mediated by inhibition of the Ca2+/CaMKII/CREB signalling pathway (Song et al., 2021).

Previous studies have reported that KOR agonists have beneficial effects in models of cognitive dysfunction in general. Takahashi et al. (2018) found that intracerebroventricular (i.c.v.) administration of U50,488 or the KOR peptide dynorphin A, reduced cognitive dysfunction in mice that had undergone excision of the olfactory bulb. Furthermore, Fan et al. (2021), Ding et al. (2021), and Li et al. (2019) all found that U50,488 mitigated postoperative cognitive dysfunction in rats that underwent cardiopulmonary bypass. Fan et al. (2021) also reported that U50,488 reduced hippocampal damage, inhibited the rate of neuronal apoptosis, and promoted recovery from oxidative stress–induced injury.

In contrast, other studies have shown that KOR agonists disrupt cognition. Abraham et al. (2021) found that dynorphin-induced KOR activation in the medial prefrontal cortex disrupted cognition in mice undergoing acute morphine withdrawal. Furthermore, U50,488 has been shown to inhibit novel object recognition in mice (Paris et al., 2011), and SalA reduced motivation and increased processing deficits in rats that were made to complete a multi-choice serial reaction time task (Nemeth et al., 2010).

Collectively, these findings indicate that KOR agonists can be both beneficial and detrimental to cognition. It remains to be determined whether KOR agonists can promote remyelination and repair in AD and preclinical models of cognitive decline, and further studies are needed to fully evaluate the role of KOR in AD.

### 3.5 Parkinson’s Disease

Parkinson’s disease is a neurological disorder that causes loss of dopaminergic neurons, predominantly in the substantia nigra resulting in movement problems such as rigidity, slowness, and tremor in more than 6 million Parkinson’s disease patients worldwide (Armstrong and Okun, 2020). Levodopa is a standard treatment for patients with Parkinson’s disease, but its side effects include dyskinesia (Pandey and Srivanitchapoom, 2017). Fortunately, there is evidence that KOR agonists may act to attenuate levodopa-induced dyskinesia. Marin et al. (2003) found that acute U50,488 administration attenuated levodopa-induced rotational behaviour in the parkinsonian rats. Cox et al. (2007) showed that U50,488 reduced levodopa-induced dyskinesia in rat and monkey models of Parkinson’s disease, but the KOR agonist also lessened levodopa’s antiparkinsonian effects. Ikeda et al. (2009) reported that nalfurafine also attenuated levodopa-induced dyskinesia in a rat model of parkinsonism. Furthermore, Hughes et al. (1998) reported that enadoline and U69,593 both increased healthy locomotor behaviours in a rat model of parkinsonism and that co-administering enadoline with levodopa reduced the doses of levodopa necessary to achieve therapeutic effects. Although studies have shown that KOR agonists can reduce dyskinesias, further studies are required to elucidate the mechanisms through which opioid compounds modulate the occurrence of L-dopa-induced dyskinesias (LIDs). A final point worth noting is that patients with Parkinson’s disease also have altered brain myelin content (Dean et al., 2016), which suggests that remyelination-promoting drugs may be helpful. Taken together, these findings indicate that KOR agonists may have a useful role as adjunct treatments for patients with Parkinson’s disease. However, neural mechanisms underlying LID in PD are still unclear and the fundamental neural connections are not well understood so exact mechanisms are still unknown.

### 3.6 Tourette’s Syndrome

Tourette’s syndrome is a neuropsychiatric disorder characterised by the presence of multiple motor and vocal tics (Du et al., 2010). It is estimated to affect between 0.3% and 0.9% of children (Scharf et al., 2015). The endogenous KOR peptide dynorphin A (1–17), is present at reduced levels in striatal fibres projecting to the globus pallidus in the postmortem brains of patients with Tourette’s syndrome (Haber et al., 1986). This finding, among others, prompted Chappell et al. (1993) to conduct a pilot study using the KOR agonist spiradoline to investigate KOR agonism as a treatment for controlling phonic and motor tics. Their results indicated that spiradoline reduced tic frequencies, but spiradoline’s unfavourable adverse event profile prevented clinical use (Wadenberg, 2003). More recently, it has been shown that in zebrafish, downregulation of the gene encoding KOR, *OPRK1*, induced a hyperkinetic phenotype in zebrafish (Depienne et al., 2019). This provided preliminary supporting data suggesting that upregulation of KOR may have a protective phenotype. While the zebrafish model provides a valuable tool for investigating genetic phenotypes, it does not fully encapsulate the complexities of this disorder. However, this study highlights that decreases in KOR expression *in vivo* could lead to an early transient hyperactivity phenotype mimicking Tourette’s syndrome. This may provide a model of Tourette’s syndrome that will enable a detailed evaluation the role of KOR and other genes in Tourette’s syndrome pathogenesis.

### 3.7 Immune-Mediated Diseases

Belkowski et al. (1995) first reported that KOR mRNA was present in immune cells (immature thymoma cell line R1.1). KOR is expressed on thymocytes (Ignatowski and Bidlack, 1998), microglia (Chao et al., 1996), macrophages (Alicea et al., 1998), and lymphocytes (Suzuki et al., 2001). The presence of KORs on so many classes of immune cells suggests that KORs play an important role in regulating immune responses.

However, the roles KOR may play in modulating the immune system are well known and is highly variable. In patients with rheumatoid arthritis, KOR mRNA was expressed in T and B cells, macrophages, and natural killer cells, but natural killer cells taken from healthy volunteers also express KOR. This led researchers to propose that, in addition to modulating nociception, KOR may play an important role in modulating anti-inflammatory effects in chronic inflammatory disorders (Gunji et al., 2000).

In studies using KOR-knockout mice, higher Ig (Ig, IgM, IgG1, and IgG2) responses have been observed, which suggests that endogenous KOR activation may induce inhibition of antibody responses (Gavériaux-Ruff et al., 2003). Other studies have shown that dynorphin increases the production of macrophage superoxide (Sharp et al., 1985), increases cytokine IL-1 production by bone marrow macrophages (Apte et al., 1990), modulates macrophage oxidative bursts (Tosk et al., 1993), and enhances macrophage tumoricidal activity (Foster and Moore, 1987).

Inflammatory diseases are often characterised by an increase in monocyte-derived cells and overproduction of IL-6 (Melnicoff et al., 1989; Ishihara and Hirano, 2002) and inhibition of IL-6 activity is a potential treatment option for many inflammatory diseases (Goldblatt and Isenberg, 2005). Parkhill and Bidlack (2006) reported that U50,488 not only inhibited the synthesis of IL-1 and TNF-α but also reduced liposaccharide-stimulated IL-6 secretion in a macrophage cell line (P388D1), and KOR antagonism blocked this effect. Paton et al. (2017) previously reported that SalA and β-THP SalB both reduce inflammatory pain, inflammation and formalin-induced oedema. SalA, ICI 204,448 and β-THP SalB also reduce neutrophil numbers in inflamed footpad tissue (Paton et al., 2017). Thus, KOR agonists may serve as a viable candidate for the treatment of immune-mediated diseases, and may provide a distinctive opportunity for the development of novel anti-inflammatory agents targeting KOR.

### 3.8 Osteoarthritis

Osteoarthritis is a progressive, disabling joint disorder that is estimated to affect almost 27 million people in the USA (Ashford and Williard, 2014). Walker et al. (1995) reported that U50,488 possessed anti-arthritic effects. In this study, subcutaneously injected U50,488 was shown to reduce the progression of arthritis in a rat model whereby the right hind paw is damaged by the administration of Freund’s adjuvant. Measures of disease severity were determined by evaluating changes in contralateral limb size, arthritis severity score, physical disability, alongside radiological and histological changes in the joint. U50,488 successfully reduced soft tissue swelling, radiographically assessed joint damage, and microscopic pathology scores.

Furthermore, other studies have reported that peripheral administration of U50,488 and asimadoline prevented joint destruction and inflammation during the onset of the disease utilising the same Freund’s adjuvant model in rats (Wilson et al., 1996; Binder and Walker, 1998). U50,488 (0.3 mg) also reduced ankle joint inflammation, hind paw oedema, and cartilage damage in arthritis models in albino Lewis rats (Bileviciute-Ljungar et al., 2006). Studies on the Freud’s adjuvant model of arthritic pain in Wistar rats have shown that U69,593 significantly reduced paw oedema and histological scores (Binder et al., 2001). Another study utilising the adjuvant arthritis model in Lewis rats showed that U50,488 significantly attenuated experimental arthritis and this attenuation was mediated through peripheral KORs in the arthritic joint (Wilson et al., 1996).

In mice lacking the KOR there were higher levels of cartilage degeneration and an increased expression of catabolic enzymes and proinflammatory cytokines following injury, while KOR activation inhibited the expression of catabolic enzymes and cartilage degradation (Wu et al., 2017). Recently, it was reported that the KOR agonist JT09 modulates the Hedgehog signalling pathway in chondrocytes from both healthy and osteoarthritic human articular chondrocytes. In this study, JT09 decreased matrix degeneration in articular chondrocytes and cartilage explants *in vivo* in rat models supporting the novel molecular mechanism for the role of the KOR in osteoarthritis (Weber et al., 2020). In a phase II clinical study, oral administration of CR845 (5 mg) exhibited significant (69%) reduction in joint pain score in patients with OA (Bagal et al., 2017). Taken together, all these studies suggest that KOR agonists may represent a striking therapeutic modality and provide an improved quality of life for patients with arthritis. KOR agonists may present a combined benefit of maintaining the functional ability of joints as well as providing antinociceptive effects in osteoarthritis. However, further studies are needed to explore the therapeutic potential of KOR agonists in preclinical and clinical models of OA.

### 3.9 Atopic Dermatitis

Atopic dermatitis is a relapsing inflammatory skin disorder with a complex pathophysiology. In atopic dermatitis there is dysregulation of both immune and nonimmune structural elements in the skin that are crucial for maintaining hydration and providing a protective barrier against pathogens, allergens, toxins, and irritants. Once this epidermal barrier is disrupted, it results in increased transepidermal water loss and increased sensitivity to external insults. Furthermore, both innate and adaptive immune systems become dysregulated and contribute to a chronic inflammatory response in keratinocytes (Egawa and Kabashima, 2018; Weidinger et al., 2018).

KORs are widely expressed on human epidermal keratinocytes (Tominaga et al., 2007; Cheng et al., 2008), dermal fibroblasts (Salemi et al., 2005; Cheng et al., 2008), mononuclear cells (Salemi et al., 2005), and subepidermal nerve fibres (Cheng et al., 2008). KORs expressed on keratinocytes play a role in keratinocyte proliferation and differentiation. In atopic dermatitis sufferers there is a downregulation of KOR expression within the epidermis (Tominaga et al., 2007), and KOR knockout mice show epidermal hypotrophy and increased cutaneous nerve fibre density in dry skin dermatitis models (Bigliardi-Qi et al., 2007). This evidence has identified KOR as a therapeutic target for inducing anti-pruritic effects and therefore a potential treatment option for patients with atopic dermatitis.

Utilizing *in vitro* cellular models, KOR activation has been shown to induce anti-inflammatory responses by down-regulating inflammatory cytokines and chemokines (Finley et al., 2008). These studies highlight that KOR plays an important role in modulating inflammatory processes in atopic dermatitis.

In the atopic dermatitis from Japanese mice (ADJM) model, orally administered nalfurafine reduced scratching behaviours (Nakasone et al., 2015). Furthermore, in a murine model of oxazolone-induced atopic dermatitis, topical application of nalfurafine also showed a significant reduction in scratching behaviours (Elliott et al., 2016). This is promising evidence as nalfurafine is already used clinically to treat forms of pruritis (Nakao and Mochizuki, 2009). This provides strong evidence that KOR agonists have potential therapeutic effects in atopic dermatitis *via* dual modulation of pruritis and inflammation.

Recently, CARA therapeutics completed a phase II clinical trial for an oral formulation of the peripherally restricted KOR agonist CR845 (difelikefalin) for the treatment of pruritus associated with patients that have atopic dermatitis (trial number NCT04018027). The most recent update on the trial involved 401 patients in a 12-week placebo controlled, randomised trial using three different concentrations of CR845. Further results are yet to be published.

Nalbuphine (Nubain) is a mixed opioid agonist-antagonist analgesic (Errick and Heel, 1983) with KOR partial-agonist and weak MOR antagonist properties (Hoskin and Hanks, 1991). Nalbuphine is also being evaluated by Trevi Therapeutics for anti-pruritic effects in the Phase 2b/3 PRISM clinical trial, scheduled to be completed in 2022. This clinical trial of 360 patients will assess nalbuphine for efficacy in providing anti-pruritic effects as well as assessment of the compound’s safety profile. Nalbuphine pre-treatment in the 1-fluro-2,4-dinitrobenzene induced model of contact dermatitis, alleviated scratching behaviours in both a time- and dose-dependent manner. Nalbuphine also increase the presence of IL-10 which is an anti-inflammatory mediator as well as increasing chemokine and cytokine levels involved in the inflammatory healing process, suggesting anti-pruritic effects (Inan et al., 2019). Furthermore, a previous clinical study involving 373 haemodialysis patients showed that nalbuphine decreased the numerical rating score of itch intensity and that patients experienced a reduction in sleep disruption (Mathur et al., 2017).

Overall, these studies indicate that KOR agonists have the potential to alter the immune environment and may provide an exciting opportunity for the therapeutic application of opioid immunopharmacology. However, more experimental work involving signal transduction pathways is needed to unravel the effects of KOR agonists on the physiological and pathological functions of the immune system.

### 3.10 Gastrointestinal Diseases

Inflammatory bowel disease (IBD) is the most commonly diagnosed gastrointestinal condition, and it causes patients to experience substantial pain and discomfort in the abdomen (Chey et al., 2015). Two widely used and well-established models of colitis are the trinitrobenzene sulfonic acid (TNBS) and dextran sodium sulfate (DSS) models which are colitic inducers.

Both asimadoline and ICI 204,488 have been shown to exert anti-nociceptive effects in the TNBS rat model of colon inflammation by producing potent inhibitory effects on the pelvic afferent nerve fibres that innervate the colon. These compounds have also proved to be effective in inhibiting visceromotor responses to induced colonic distension which is a well-established model of visceral pain (Sengupta et al., 1999). Administration of the selective KOR agonist SalA attenuated the effects of TNBS and DSS induced preclinical models of colitis producing potent anti-inflammatory and anti-nociceptive effects. These effects were shown to be mediated through both KOR and cannabinoid receptors (CB1). This effect may occur through the formation of KOR/CB1heterodimers. SalA showed anti-inflammatory effects which the authors suggest may work through both neural and immune mediated mechanisms simultaneously. Previous research has highlighted ultrapotent actions on macrophages by reducing the release of inflammatory mediators. Thus, showing anti-inflammatory effects *in vitro* (Aviello et al., 2011; Fichna et al., 2012). However, SalA is not a desirable clinical candidate due to hallucinogenic effects and a short duration of action (Ranganathan et al., 2012).

Furthermore, PR-38 is a structurally novel analogue of SalA developed to have an improved pharmacological profile which has increased safety and efficacy. PR-38 acts as an agonist at KOR, MOR and CB1 receptors and has improved oral bioavailability. This compound was found to have potent anti-nociceptive effects mediated *via* MOR and anti-inflammatory effects *via* KOR in a preclinical model of colitis. In this study, behavioural nociceptive responses to intracolonic mustard oil (1%) were assessed in control and trinitrobenzene sulfonic acid (TNBS)-treated mice. The instillation of mustard oil in control mice significantly increased the number of postures defined as spontaneous nociceptive-related behaviours and administration of PR-38 (10 mg/kg/i.p.) decreased the number of nociceptive responses (Sałaga et al., 2014). Administration of PR-38 *in vitro* significantly inhibited colonic motility, increased gastrointestinal transit time, and reversed hypermotility in models of gastrointestinal disorders. PR-38 (10 mg/kg/i.p.) showed improved side effects with no changes in spontaneous locomotor activity believed to be due to reduced blood brain barrier penetration (Sałaga et al., 2014).

P-317 is a novel analogue of the opioid peptide morphiceptin and has mixed MOR and KOR agonist activity (Sobczak et al., 2014). P-317 (0.1 mg/kg, i.p; 1 mg/kg, p.o) was shown to attenuate mustard oil-induced nociceptive behaviours in TNBS-treated mice. P-317 also decreased mRNA expression of pro-inflammatory cytokines and improved the ulcer score and colon length in mouse models of IBD and Crohn’s disease when administered both peripherally and orally at low doses (Sobczak et al., 2014). More recently P-317 was also shown to repair damage in models of colitis in mice (Zielińska et al., 2020).

Similarly, Sialorphin is a peptide that has previously shown anti-nociceptive effects in preclinical models of acute pain. More recently, it has shown to be effective in attenuating colitis in the TNBS and DSS preclinical models with potent anti-inflammatory effects (Salaga et al., 2017). This data provides promising evidence of the therapeutic potential of KOR agonists for the treatment of IBD and associated gastrointestinal disorders through their ability to produce both anti-nociceptive and immunomodulatory effects.

In clinical trials KOR agonist administration has also been shown to be effective in modulating symptoms of irritable bowel syndrome (IBS), which is characterised by changes in bowel habits and abdominal pain and discomfort (Chey et al., 2015). Administration of asimadoline in a phase II clinical trial provided analgesia and relieved discomfort for 596 patients with IBS, significantly improving their symptoms. Adverse effects such as diarrhoea, abdominal pain, vomiting and nausea were associated with administration, but the rate of discontinuation due to these effects were low (Mangel et al., 2008). Phase III clinical trials for asimadoline administration were completed in 2013, however, these results have not yet been published. Following this, the peripheral KOR agonist, fedotozine was found to be effective in reducing both disease severity and pain in patients with IBS by reducing symptoms of abdominal pain and bloating. Throughout this phase II clinical investigation, fedotozine was administered three times daily, was well tolerated by patients, and had a good clinical safety profile (Dapoigny et al., 1995) (See Tables 1, 2 for details).

Furthermore, opioid receptors have now been identified as a potential therapeutic target for the treatment of food allergy. Opioid receptors, specifically KOR and MOR, are widely expressed throughout the gastrointestinal tract and function to regulate gut motility and gastrointestinal transit. In a well-established mouse model of ovalbumin induced allergic diarrhoea, pre-treatment with the KOR agonist U50,488 significantly improved disease severity measured through clinical macroscopic scores and alleviated ovalbumin induced symptoms including diarrhoea, increased plasma mouse mast cell protease 1 (MMCP-1) and IgE levels, mastocytosis and Th2 intestinal responses. In this model, U50,488 induced a decreased in mast cell numbers within the small intestinal mucosa and plasma MMCP-1 concentrations quantified from extracted blood samples. This suggests that KOR signalling is involved in murine allergic diarrhoea and their role is beyond the anti-diarrheal effects and involves the modulation of mucosal immune responses associated with food allergy. However, further experiments are needed to determine the effects of KORs in modulating the immune parameters in food allergy (Duncker et al., 2012).

### 3.11 Cancer

There is evidence to show that KOR agonists modulate angiogenesis and may have beneficial effects in modulating tumour growth. Yamamizu et al. (2011) demonstrated that KOR agonists U50,488 and nalfurafine inhibited the expression of vascular EGFR-2 (VEGFR2) and induced upregulation of anti-tumour angiogenic modulators. KOR agonism inhibited tumour angiogenesis by suppressing vascular endothelial growth factor signalling during vascular differentiation and tumorigenesis *via* inhibition of the cyclic adenosine monophosphate (cAMP)/protein kinase A signalling pathway (Yamamizu et al., 2011; Yamamizu et al., 2013). KOR agonists U50,488 and nalfurafine were also shown to inhibit human umbilical vein endothelial cell migration and vascular tube formation by suppressing VEGFR2 expression. Intraperitoneal injections of a low dose of nalfurafine not only reduced tumour sizes but also inhibited tumour angiogenesis specifically through KOR activation (Yamamizu et al., 2013). In another study, U50,488 dose-dependently decreased tumour cell growth in lung cancer cell lines through the phosphorylated-glycogen synthase kinase 3β signalling pathway (Kuzumaki et al., 2012). A study by Chen et al. (2017) detected KOR mRNA in human liver cancer cells and demonstrated that KOR mRNA expression was lower in cancerous cells than in adjacent normal tissue. This study reported that patients with downregulated KOR within cancerous liver cells had a reduced survival rate and increased recurrence. This suggests that KOR might have some tumour suppressing effects in liver cancer. Because cancer patients also require pain relief, and KORs modulate pain, there is therapeutic potential for developing novel cancer treatment strategies whereby KOR agonists may provide pain relief and anti-tumour effects. However, the literature on how KOR activation modulates cancer proliferation and tumour growth is very limited. Further studies are needed to test the utility of KOR agonists and to understand the mechanism of action within various cancer types. For a recent review on the therapeutic potential of KOR agonists in cancer see Zhou et al. (2022).

### 3.12 Hypoxia, Ischemia, and Cardiac Dysfunction

Ischemia is a state of restricted or insufficient blood flow to part of the body, and the localised lack of oxygen leads to tissue hypoxia. In a mouse model of stroke, Guéniau and Oberlander (1997) found that the KOR agonist niravoline reduced brain oedema. Similarly, Chen et al. (2005) found that the selective KOR agonist BRL-52537 provided ischemic neuroprotection in male (but not female) rats. Similar studies showed that KOR agonists BRL-52537 (Fang et al., 2013), SalA (Xin et al., 2016), and U50,488 (Charron et al., 2008) had neuroprotective effects in various rat models of ischemia. More recently, using a mouse model of chronic hypoxia-induced brain injury, recapitulating hypoxic brain injury seen in preterm infants, U50,488 was shown to promote oligodendroglia differentiation and remyelination. KOR agonism was shown to rescue synapse numbers and facilitate the recovery of motor and cognitive functions (Wang et al., 2018). The enhanced remyelination in this model was suggested to be of therapeutic benefit to preterm infants with hypoxia-related white matter injury, a condition that occurs in 5%–10% of preterm infants (Wang et al., 2018).

KOR is highly expressed in the mouse heart (Giros et al., 1995). In a rat model of myocardial ischemia and reperfusion injury, administration of U50,488 decreased the incidence and duration of various forms of arrhythmia, such as premature ventricular contractions, ventricular tachycardia, and ventricular fibrillation (Jin-Cheng et al., 2008). Activation of KORs by U50,488 has also been shown to attenuate cardiomyocyte apoptosis by inhibiting caspase activity and Bcl/Bax protein levels (Rong et al., 2009). Studies have shown that U50,488 inhibits TNF-α production and reduces neutrophil infiltration into the ischemic/reperfused myocardial tissue, thereby reducing damage to myocardial tissue *via* cardioprotective and anti-inflammatory mechanisms. U50,488 modulated Toll-like receptor 4 (TLR4) and nuclear factor kappa-light-chain-enhancer of activated B cells (NF-κB) (Wu et al., 2011; Lin et al., 2013). Administration of U50,488 improved cardiac function and neovascularisation following myocardial ischemia and reperfusion injury in rats, which results in a reduced myocardial infarct size and reductions in oxidative stress, hypertrophy, and fibrosis (Tong et al., 2016). Xin et al. (2016) found that SalA promoted cognitive recovery in a rat model of forebrain ischemia, and Charron et al. (2008) showed that U50,488 mitigated spatial memory deficits in rats following global ischemia.

These studies, utilising multiple preclinical models of hypoxic damage/ischemia/reperfusion injury, clearly show that KOR activation has beneficial effects in protecting against hypoxic brain injury and cardiac ischemia *via* multiple mechanisms. Interestingly, KOR agonist-induced remyelination and repair in hypoxic brain injury has a mechanism of action similar to that seen in preclinical MS models, notably promoting remyelination and repair *via* OPC differentiation. However, given KOR’s ability to reduce inflammation, additional mechanisms cannot be ruled out and require further investigation.

## 4 Conclusion

KOR is a promising target for drug discovery and development efforts. KOR regulates numerous intracellular signalling pathways and myriad physiological processes, including stress, mood, reward, pain, the immune system, angiogenesis and remyelination. Pharmacological investigations to date have yielded evidence that KOR agonists may be useful as treatments for chronic pain, pruritis, multiple sclerosis, AD, immune modulated diseases, gastrointestional diseases, cancer, hypoxia and ischemia and various other disorders. Notably, KOR agonists are free of the abuse potential and respiratory depression associated with MOR agonists. However, despite considerable drug-development efforts, traditional KOR agonists have failed to provide clinical drugs, largely due to unfavourable side effects. More recent drug-development efforts described in this review have utilised a wide variety of structural scaffolds to develop novel compounds targeting KOR with clinical potential and improved side-effects. The clinical use and favourable safety of nalfurafine (Remitch) in Japan, and recent FDA approval of difelikefalin (Korsuva) in the US provide examples of the clinical potential of KOR agonists. One of the biggest challenges in drug discovery is to model human conditions in animals. Animal studies are critical for understanding the biology and pathophysiology of diseases, but they do not guarantee clinical success.

Development of KOR agonists as clinical therapeutics is not without significant challenges. We have provided examples of three strategies commonly utilised in attempts to overcome side effects. We have presented studies using G-protein biased agonists to reduce β-arrestin-2-dependent aversive and sedative effects. However, effective implementation of this strategy will depend on further research into the complex signalling pathways associated with KOR activation and identification of cell-signalling pathways responsible for each therapeutic application and each side-effect. The development of peripherally restricted KOR agonists has created KOR agonists without CNS-mediated side-effects. However, the limitation of this strategy is that CNS penetration is required for many diseases for which KOR agonists are being explored as therapeutics. Mixed opioid agonism is another strategy, particularly for developing pain medications largely due to the ability of KOR agonists to negate the rewarding properties of MOR agonists and potentiate the analgesic effects. This review provides compelling evidence that KOR agonists have the potential to be utilized in the clinic, and that each KOR agonist is unique in its ability to differentially regulate multiple therapeutic effects and side effects. Developing KOR agonists with an improved therapeutic index will be key to their clinical success.

## References

[B1] Abraham A. D. Casello S. M. Schattauer S. S. Wong B. A. Mizuno G. O. Mahe K. (2021). Release of Endogenous Dynorphin Opioids in the Prefrontal Cortex Disrupts Cognition. Neuropsychopharmacology 46, 2330–2339. 10.1038/s41386-021-01168-2 34545197 PMC8580977

[B2] Aceves M. Mathai B. B. Hook M. A. (2016). Evaluation of the Effects of Specific Opioid Receptor Agonists in a Rodent Model of Spinal Cord Injury. Spinal Cord. 54, 767–777. 10.1038/sc.2016.28 26927293 PMC5009008

[B3] Addy P. H. (2012). Acute and Post-acute Behavioral and Psychological Effects of Salvinorin A in Humans. Psychopharmacol. Berl. 220, 195–204. 10.1007/s00213-011-2470-6 21901316

[B4] Albert‐Vartanian A. Boyd M. R. Hall A. L. Morgado S. J. Nguyen E. Nguyen V. P. H. (2016). Will Peripherally Restricted Kappa‐opioid Receptor Agonists (pKORA S) Relieve Pain with Less Opioid Adverse Effects and Abuse Potential? J. Clin. Pharm. Ther. 41, 371–382. 27245498 10.1111/jcpt.12404

[B5] Alicea C. Belkowski S. M. Sliker J. K. Zhu J. Liu-Chen L. Y. Eisenstein T. K. (1998). Characterization of Kappa-Opioid Receptor Transcripts Expressed by T Cells and Macrophages. J. Neuroimmunol. 91, 55–62. 10.1016/s0165-5728(98)00151-9 9846819

[B6] Allied Market Research (2020). Global Pain Management Drugs Market: Opportunities and Forecast, 2020–2027.

[B7] Apte R. N. Durum S. K. Oppenheim J. J. (1990). Opioids Modulate Interleukin-1 Production and Secretion by Bone-Marrow Macrophages. Immunol. Lett. 24, 141–148. 10.1016/0165-2478(90)90026-m 2162329

[B8] Arendt-Nielsen L. Olesen A. E. Staahl C. Menzaghi F. Kell S. Wong G. Y. (2009). Analgesic Efficacy of Peripheral Kappa-Opioid Receptor Agonist CR665 Compared to Oxycodone in a Multi-Modal, Multi-Tissue Experimental Human Pain Model: Selective Effect on Visceral Pain. Anesthesiology 111, 616–624. 10.1097/ALN.0b013e3181af6356 19672186

[B9] Argyriou A. A. Makris N. (2008). Neuromyelitis Optica: a Distinct Demyelinating Disease of the Central Nervous System. Acta Neurol. Scand. 118, 209–217. 10.1111/j.1600-0404.2008.01002.x 18336627

[B10] Armstrong M. J. Okun M. S. (2020). Diagnosis and Treatment of Parkinson Disease: A Review. JAMA 323, 548–560. 10.1001/jama.2019.22360 32044947

[B11] Arvanitakis Z. Shah R. C. Bennett D. A. (2019). Diagnosis and Management of Dementia: Review. JAMA 322, 1589–1599. 10.1001/jama.2019.4782 31638686 PMC7462122

[B12] Ashford S. Williard J. (2014). Osteoarthritis: a Review. Nurse Pract. 39, 1–8. 10.1097/01.NPR.0000445886.71205.c4 24739424

[B13] Aspli K. T. Flaten T. P. Roos P. M. Holmøy T. Skogholt J. H. Aaseth J. (2015). Iron and Copper in Progressive Demyelination--New Lessons from Skogholt's Disease. J. Trace Elem. Med. Biol. 31, 183–187. 10.1016/j.jtemb.2014.12.002 25563774

[B14] Atigari D. V. Paton K. F. Uprety R. Váradi A. Alder A. F. Scouller B. (2021). The Mixed Kappa and Delta Opioid Receptor Agonist, MP1104, Attenuates Chemotherapy-Induced Neuropathic Pain. Neuropharmacology 185, 108445. 10.1016/j.neuropharm.2020.108445 33383089 PMC8344368

[B15] Aubourg P. (2015). Cerebral Adrenoleukodystrophy: a Demyelinating Disease that Leaves the Door Wide Open. Brain 138, 3133–3136. 10.1093/brain/awv271 26503938

[B16] Aviello G. Borrelli F. Guida F. Romano B. Lewellyn K. de Chiaro M. (2011). Ultrapotent Effects of Salvinorin A, a Hallucinogenic Compound from Salvia Divinorum, on LPS-Stimulated Murine Macrophages and its Anti-inflammatory Action *In Vivo* . J. Mol. Med. Berl. 89, 891–902. 10.1007/s00109-011-0752-4 21499737

[B17] Bagal M. C. S. Brady P. Stauffer J. (2017). “A Phase 2, Randomized, Double-Blind, Placebo-Controlled, Titration-To-Effect Study of Orally Administered CR845 in Patients with Osteoarthritis of the Hip or Knee. ACR,” in ARHP Annual Meeting. *Arthritis Rheumatol* .

[B18] Bagnol D. Mansour A. Akil H. Watson S. J. (1995). Localization of Mu and Kappa Opioid Receptors in Rat Colon by Antibodies to the Cloned Opioid Receptors. Analg. 1, 264–267. 10.3727/107156995819563267

[B19] Barber A. Bartoszyk G. D. Bender H. M. Gottschlich R. Greiner H. E. Harting J. (1994). A Pharmacological Profile of the Novel, Peripherally-Selective Kappa-Opioid Receptor Agonist, EMD 61753. Br. J. Pharmacol. 113, 1317–1327. 10.1111/j.1476-5381.1994.tb17142.x 7889287 PMC1510549

[B20] Barber A. Gottschlich R. (1997). Novel Developments with Selective, Non-peptidic Kappa-Opioid Receptor Agonists. Expert Opin. Investig. Drugs 6, 1351–1368. 10.1517/13543784.6.10.1351 15989506

[B21] Beck T. C. Dix T. A. (2019). Targeting Peripheral ϰ-opioid Receptors for the Non-addictive Treatment of Pain. Future Drug Discov. 1. 10.4155/fdd-2019-0022 PMC6859935 31742251

[B22] Beck T. C. Reichel C. M. Helke K. L. Bhadsavle S. S. Dix T. A. (2019). Non-addictive Orally-Active Kappa Opioid Agonists for the Treatment of Peripheral Pain in Rats. Eur. J. Pharmacol. 856, 172396. 10.1016/j.ejphar.2019.05.025 31103632 PMC6696947

[B23] Bedini A. di Cesare Mannelli L. Micheli L. Baiula M. Vaca G. de Marco R. (2020). Functional Selectivity and Antinociceptive Effects of a Novel Kopr Agonist. Front. Pharmacol. 11. 10.3389/fphar.2020.00188 PMC7066533 32210803

[B24] Belkowski S. M. Zhu J. Liu-Chen L. Y. Eisenstein T. K. Adler M. W. Rogers T. J. (1995). Sequence of Kappa-Opioid Receptor cDNA in the R1.1 Thymoma Cell Line. J. Neuroimmunol. 62, 113–117. 10.1016/0165-5728(95)00116-j 7499487

[B25] Bigliardi-Qi M. Gaveriaux-Ruff C. Pfaltz K. Bady P. Baumann T. Rufli T. (2007). Deletion of Mu- and Kappa-Opioid Receptors in Mice Changes Epidermal Hypertrophy, Density of Peripheral Nerve Endings, and Itch Behavior. J. Invest. Dermatol 127, 1479–1488. 10.1038/sj.jid.5700661 17185983

[B26] Bileviciute-Ljungar I. Saxne T. Spetea M. (2006). Anti-inflammatory Effects of Contralateral Administration of the Kappa-Opioid Agonist U-50,488H in Rats with Unilaterally Induced Adjuvant Arthritis. Rheumatol. Oxf. 45, 295–302. 10.1093/rheumatology/kei156 16249243

[B27] Binder W. Machelska H. Mousa S. Schmitt T. Rivière P. J. Junien J. L. (2001). Analgesic and Antiinflammatory Effects of Two Novel Kappa-Opioid Peptides. Anesthesiology 94, 1034–1044. 10.1097/00000542-200106000-00018 11465595

[B28] Binder W. Walker J. S. (1998). Effect of the Peripherally Selective Kappa-Opioid Agonist, Asimadoline, on Adjuvant Arthritis. Br. J. Pharmacol. 124, 647–654. 10.1038/sj.bjp.0701874 9690855 PMC1565434

[B29] Birch P. J. Rogers H. Hayes A. G. Hayward N. J. Tyers M. B. Scopes D. I. (1991). Neuroprotective Actions of GR89696, a Highly Potent and Selective Kappa-Opioid Receptor Agonist. Br. J. Pharmacol. 103, 1819–1823. 10.1111/j.1476-5381.1991.tb09869.x 1657267 PMC1907793

[B30] Bohn L. M. Aubé J. (2017). Seeking (And Finding) Biased Ligands of the Kappa Opioid Receptor. ACS Med. Chem. Lett. 8, 694–700. 10.1021/acsmedchemlett.7b00224 28740600 PMC5512133

[B31] Bonaz B. Rivière P. J. Sinniger V. Pascaud X. Junien J. L. Fournet J. (2000). Fedotozine, a Kappa-Opioid Agonist, Prevents Spinal and Supra-spinal Fos Expression Induced by a Noxious Visceral Stimulus in the Rat. Neurogastroenterol. Motil. 12, 135–147. 10.1046/j.1365-2982.2000.00188.x 10771495

[B32] Bouhrara M. Reiter D. A. Bergeron C. M. Zukley L. M. Ferrucci L. Resnick S. M. (2018). Evidence of Demyelination in Mild Cognitive Impairment and Dementia Using a Direct and Specific Magnetic Resonance Imaging Measure of Myelin Content. Alzheimers Dement. 14, 998–1004. 10.1016/j.jalz.2018.03.007 29679574 PMC6097903

[B33] Bove R. M. Green A. J. (2017). Remyelinating Pharmacotherapies in Multiple Sclerosis. Neurotherapeutics 14, 894–904. 10.1007/s13311-017-0577-0 28948533 PMC5722779

[B34] Braida D. Donzelli A. Martucci R. Capurro V. Sala M. (2011). Learning and Memory Impairment Induced by Salvinorin A, the Principal Ingredient of Salvia Divinorum, in Wistar Rats. Int. J. Toxicol. 30, 650–661. 10.1177/1091581811418538 21960665

[B35] Briggs S. L. Rech R. H. Sawyer D. C. (1998). Kappa Antinociceptive Activity of Spiradoline in the Cold-Water Tail-Flick Assay in Rats. Pharmacol. Biochem. Behav. 60, 467–472. 10.1016/s0091-3057(98)00017-3 9632230

[B36] Brooks D. P. Giardina G. Gellai M. Dondio G. Edwards R. M. Petrone G. (1993). Opiate Receptors within the Blood-Brain Barrier Mediate Kappa Agonist-Induced Water Diuresis. J. Pharmacol. Exp. Ther. 266, 164–171. 8392549

[B37] Bruchas M. R. Chavkin C. (2010). Kinase Cascades and Ligand-Directed Signaling at the Kappa Opioid Receptor. Psychopharmacol. Berl. 210, 137–147. 10.1007/s00213-010-1806-y PMC3671863 20401607

[B38] Brust T. F. Morgenweck J. Kim S. A. Rose J. H. Locke J. L. Schmid C. L. (2016). Biased Agonists of the Kappa Opioid Receptor Suppress Pain and Itch without Causing Sedation or Dysphoria. Sci. Signal 9, ra117. 10.1126/scisignal.aai8441 27899527 PMC5231411

[B39] Butelman E. R. Prisinzano T. E. Deng H. Rus S. Kreek M. J. (2009). Unconditioned Behavioral Effects of the Powerful Kappa-Opioid Hallucinogen Salvinorin A in Nonhuman Primates: Fast Onset and Entry into Cerebrospinal Fluid. J. Pharmacol. Exp. Ther. 328, 588–597. 10.1124/jpet.108.145342 19001155 PMC2682281

[B40] Butelman E. R. Vivian J. A. Yu J. Kreek M. J. Woods J. H. (1999). Systemic Effects of E-2078, a Stabilized Dynorphin A(1-8) Analog, in Rhesus Monkeys. Psychopharmacol. Berl. 143, 190–196. 10.1007/s002130050935 10326782

[B41] Caram-Salas N. L. Reyes-García G. Bartoszyk G. D. Araiza-Saldaña C. I. Ambriz-Tututi M. Rocha-González H. I. (2007). Subcutaneous, Intrathecal and Periaqueductal Grey Administration of Asimadoline and ICI-204448 Reduces Tactile Allodynia in the Rat. Eur. J. Pharmacol. 573, 75–83. 10.1016/j.ejphar.2007.06.034 17643411

[B42] Carlezon W. A. JR. Béguin C. Dinieri J. A. Baumann M. H. Richards M. R. Todtenkopf M. S. (2006). Depressive-like Effects of the Kappa-Opioid Receptor Agonist Salvinorin A on Behavior and Neurochemistry in Rats. J. Pharmacol. Exp. Ther. 316, 440–447. 10.1124/jpet.105.092304 16223871

[B43] Chang Q. Hanania T. Mash D. C. Maillet E. L. (2015). Noribogaine Reduces Nicotine Self-Administration in Rats. J. Psychopharmacol. 29, 704–711. 10.1177/0269881115584461 25995321 PMC4456428

[B44] Chao C. C. Gekker G. Hu S. Sheng W. S. Shark K. B. Bu D. F. (1996). Kappa Opioid Receptors in Human Microglia Downregulate Human Immunodeficiency Virus 1 Expression. Proc. Natl. Acad. Sci. U. S. A. 93, 8051–8056. 10.1073/pnas.93.15.8051 8755601 PMC38873

[B45] Chappell P. B. Leckman J. F. Scahill L. D. Hardin M. T. Anderson G. Cohen D. J. (1993). Neuroendocrine and Behavioral Effects of the Selective Kappa Agonist Spiradoline in Tourette's Syndrome: a Pilot Study. Psychiatry Res. 47, 267–280. 10.1016/0165-1781(93)90084-t 8396784

[B46] Charron C. Messier C. Plamondon H. (2008). Neuroprotection and Functional Recovery Conferred by Administration of Kappa- and Delta 1-opioid Agonists in a Rat Model of Global Ischemia. Physiol. Behav. 93, 502–511. 10.1016/j.physbeh.2007.10.015 18031772

[B47] Chen C. H. Toung T. J. Hurn P. D. Koehler R. C. Bhardwaj A. (2005). Ischemic Neuroprotection with Selective Kappa-Opioid Receptor Agonist Is Gender Specific. Stroke 36, 1557–1561. 10.1161/01.STR.0000169928.76321.3d 15933260

[B48] Chen D. Chen Y. Yan Y. Pan J. Xing W. Li Q. (2017). Down-regulation of the Tumour Suppressor κ-opioid Receptor Predicts Poor Prognosis in Hepatocellular Carcinoma Patients. BMC Cancer 17, 553. 10.1186/s12885-017-3541-9 28821282 PMC5562986

[B49] Cheng B. Liu H. W. Fu X. B. Sheng Z. Y. Li J. F. (2008). Coexistence and Upregulation of Three Types of Opioid Receptors, Mu, Delta and Kappa, in Human Hypertrophic Scars. Br. J. Dermatol 158, 713–720. 10.1111/j.1365-2133.2008.08449.x 18284397

[B50] Chey W. D. Kurlander J. Eswaran S. (2015). Irritable Bowel Syndrome: a Clinical Review. JAMA 313, 949–958. 10.1001/jama.2015.0954 25734736

[B51] Chu L. F. Clark D. J. Angst M. S. (2006). Opioid Tolerance and Hyperalgesia in Chronic Pain Patients after One Month of Oral Morphine Therapy: a Preliminary Prospective Study. J. Pain 7, 43–48. 10.1016/j.jpain.2005.08.001 16414554

[B52] Chung P. C. Boehrer A. Stephan A. Matifas A. Scherrer G. Darcq E. (2015). Delta Opioid Receptors Expressed in Forebrain GABAergic Neurons Are Responsible for SNC80-Induced Seizures. Behav. Brain Res. 278, 429–434. 10.1016/j.bbr.2014.10.029 25447299 PMC4382405

[B53] Coffeen U. Canseco-Alba A. Simón-Arceo K. Almanza A. Mercado F. León-Olea M. (2018). Salvinorin A Reduces Neuropathic Nociception in the Insular Cortex of the Rat. Eur. J. Pain 22, 311–318. 10.1002/ejp.1120 28975684

[B54] Cowan A. Gmerek D. E. (1986). *In-vivo* Studies on Kappa Opioid Receptors. Trends Pharmacol. Sci. 7, 69–72. 10.1016/0165-6147(86)90257-9

[B55] Cowan A. Kehner G. B. Inan S. (2015). “Targeting Itch with Ligands Selective for κ Opioid Receptors,” in Handbook of Experimental Pharmacology. 10.1007/978-3-662-44605-8_16 25861786

[B56] Cox H. Togasaki D. M. Chen L. Langston J. W. di Monte D. A. Quik M. (2007). The Selective Kappa-Opioid Receptor Agonist U50,488 Reduces L-Dopa-Induced Dyskinesias but Worsens Parkinsonism in MPTP-Treated Primates. Exp. Neurol. 205, 101–107. 10.1016/j.expneurol.2007.01.024 17335811 PMC2001245

[B57] Dapoigny M. Abitbol J. L. Fraitag B. (1995). Efficacy of Peripheral Kappa Agonist Fedotozine versus Placebo in Treatment of Irritable Bowel Syndrome. A Multicenter Dose-Response Study. Dig. Dis. Sci. 40, 2244–2249. 10.1007/BF02209014 7587797

[B58] Dean D. C. Sojkova J. Hurley S. Kecskemeti S. Okonkwo O. Bendlin B. B. (2016). Alterations of Myelin Content in Parkinson's Disease: a Cross-Sectional Neuroimaging Study. PLoS One 11, e0163774. 10.1371/journal.pone.0163774 27706215 PMC5051727

[B59] Deeks E. D. (2021). Difelikefalin: First Approval. Drugs 81, 1937–1944. 10.1007/s40265-021-01619-6 34674115

[B60] Delay-Goyet P. Zajac J.-M. Javoy-Agid F. Agid Y. Roques B. P. (1987). Regional Distribution of μ, δ and κ Opioid Receptors in Human Brains from Controls and Parkinsonian Subjects. Brain Res. 414 (1), 8–14. 10.1016/0006-8993(87)91321-7 3040166

[B61] Delvaux M. (2001). Pharmacology and Clinical Experience with Fedotozine. Expert Opin. Investig. Drugs 10, 97–110. 10.1517/13543784.10.1.97 11116283

[B62] Denny L. Al Abadey A. Robichon K. Templeton N. Prisinzano T. E. Kivell B. M. (2021). Nalfurafine Reduces Neuroinflammation and Drives Remyelination in Models of CNS Demyelinating Disease. Clin. Transl. Immunol. 10, e1234. 10.1002/cti2.1234 PMC7811802 33489124

[B63] Depienne C. Ciura S. Trouillard O. Bouteiller D. Leitao E. Nava C. (2019). Association of Rare Genetic Variants in Opioid Receptors with Tourette Syndrome. Tremor Other Hyperkinet Mov. (N Y) 9. 10.5334/tohm.464 PMC6878848 31824749

[B64] DiMattio K. M. (2016). Studies on Ligands of the Kappa Opioid Receptor. Temple University. Ph.D.

[B65] Ding G. Li D. Sun Y. Chen K. Song D. (2021). κ-Opioid Receptor Agonist Ameliorates Postoperative Neurocognitive Disorder by Activating the Ca2+/CaMKII/CREB Pathway. J. Healthc. Eng. 2021, 3401654. 10.1155/2021/3401654 34608407 PMC8487382

[B66] Dobson R. Giovannoni G. (2019). Multiple Sclerosis - a Review. Eur. J. Neurol. 26, 27–40. 10.1111/ene.13819 30300457

[B67] Dong H. P. Zhou W. Ma X. X. He Z. Z. Wang Z. H. (2018). Salvinorin A Preserves Cerebral Pial Artery Autoregulation after Forebrain Ischemia via the PI3K/AKT/cGMP Pathway. Braz J. Med. Biol. Res. 51, e6714. 10.1590/1414-431X20176714 29561955 PMC5875901

[B68] Du C. Duan Y. Wei W. Cai Y. Chai H. Lv J. (2016). Kappa Opioid Receptor Activation Alleviates Experimental Autoimmune Encephalomyelitis and Promotes Oligodendrocyte-Mediated Remyelination. Nat. Commun. 7, 11120. 10.1038/ncomms11120 27040771 PMC4822006

[B69] Du J.-C. Chiu T.-F. Lee K.-M. Wu H.-L. Yang Y.-C. Hsu S.-Y. (2010). Tourette Syndrome in Children: An Updated Review. Pediatr. Neonatol. 51, 255–264. 10.1016/s1875-9572(10)60050-2 20951354

[B70] Duncker S. C. Philippe D. Martin-Paschoud C. Moser M. Mercenier A. Nutten S. (2012). *Nigella Sativa* (Black Cumin) Seed Extract Alleviates Symptoms of Allergic Diarrhea in Mice, Involving Opioid Receptors. PLoS One 7, e39841. 10.1371/journal.pone.0039841 22768141 PMC3387213

[B71] Dunn A. D. Reed B. Erazo J. Ben-Ezra A. Kreek M. J. (2019). Signaling Properties of Structurally Diverse Kappa Opioid Receptor Ligands: toward *In Vitro* Models of *In Vivo* Responses. ACS Chem. Neurosci. 10, 3590–3600. 10.1021/acschemneuro.9b00195 31313902

[B72] Dykstra L. A. Gmerek D. E. Winger G. Woods J. H. (1987). Kappa Opioids in Rhesus Monkeys. I. Diuresis, Sedation, Analgesia and Discriminative Stimulus Effects. J. Pharmacol. Exp. Ther. 242, 413–420. 3612543

[B73] Edwards K. A. Havelin J. J. Mcintosh M. I. Ciccone H. A. Pangilinan K. Imbert I. (2018). A Kappa Opioid Receptor Agonist Blocks Bone Cancer Pain without Altering Bone Loss, Tumor Size, or Cancer Cell Proliferation in a Mouse Model of Cancer-Induced Bone Pain. J. Pain 19, 612–625. 10.1016/j.jpain.2018.01.002 29371114

[B74] Egawa G. Kabashima K. (2018). Barrier Dysfunction in the Skin Allergy. Allergol. Int. 67, 3–11. 10.1016/j.alit.2017.10.002 29153780

[B75] Ehrich J. M. Messinger D. I. Knakal C. R. Kuhar J. R. Schattauer S. S. Bruchas M. R. (2015). Kappa Opioid Receptor-Induced Aversion Requires P38 MAPK Activation in VTA Dopamine Neurons. J. Neurosci. 35, 12917–12931. 10.1523/JNEUROSCI.2444-15.2015 26377476 PMC4571610

[B76] Eisenach J. C. Carpenter R. Curry R. (2003). Analgesia from a Peripherally Active Kappa-Opioid Receptor Agonist in Patients with Chronic Pancreatitis. Pain 101, 89–95. 10.1016/s0304-3959(02)00259-2 12507703

[B77] Eisenstein T. K. (2019). The Role of Opioid Receptors in Immune System Function. Front. Immunol. 10, 2904. 10.3389/fimmu.2019.02904 31921165 PMC6934131

[B78] Elliott G. Vanwersch R. Soeberdt M. Metze D. Lotts T. Ständer S. (2016). Topical Nalfurafine Exhibits Anti-inflammatory and Anti-pruritic Effects in a Murine Model of AD. J. Dermatol Sci. 84, 351–354. 10.1016/j.jdermsci.2016.09.008 27665389

[B79] Emmerson P. J. Liu M. R. Woods J. H. Medzihradsky F. (1994). Binding Affinity and Selectivity of Opioids at Mu, Delta and Kappa Receptors in Monkey Brain Membranes. J. Pharmacol. Exp. Ther. 271, 1630–1637. 7996478

[B80] Endoh T. Matsuura H. Tajima A. Izumimoto N. Tajima C. Suzuki T. (1999). Potent Antinociceptive Effects of TRK-820, a Novel Kappa-Opioid Receptor Agonist. Life Sci. 65, 1685–1694. 10.1016/s0024-3205(99)00417-8 10573186

[B81] Endoh T. Tajima A. Suzuki T. Kamei J. Narita M. Tseng L. (2000). Characterization of the Antinociceptive Effects of TRK-820 in the Rat. Eur. J. Pharmacol. 387, 133–140. 10.1016/s0014-2999(99)00815-8 10650153

[B82] Errick J. K. Heel R. C. (1983). Nalbuphine A Preliminary Review of its Pharmacological Properties and Therapeutic Efficacy. Drugs 26, 191–211. 10.2165/00003495-198326030-00002 6137354

[B83] Escudero-Lara A. Cabañero D. Maldonado R. (2021). Kappa Opioid Receptor Modulation of Endometriosis Pain in Mice. Neuropharmacology 195, 108677. 10.1016/j.neuropharm.2021.108677 34153313

[B84] European Medicines Agency (2013). Assessment Report: Winfuran.

[B85] Ewald A. W. M. Bosch P. J. Culverhouse A. Crowley R. S. Neuenswander B. Prisinzano T. E. (2017). The C-2 Derivatives of Salvinorin A, Ethoxymethyl Ether Sal B and β-tetrahydropyran Sal B, Have Anti-cocaine Properties with Minimal Side Effects. Psychopharmacol. Berl. 234, 2499–2514. 10.1007/s00213-017-4637-2 PMC5542847 28536865

[B86] Fan J. Li L. Qu P. Diao Y. Sun Y. (2021). κ‑opioid Receptor Agonist U50488H Attenuates Postoperative Cognitive Dysfunction of Cardiopulmonary Bypass Rats through the PI3K/AKT/Nrf2/HO-1 Pathway. Mol. Med. Rep. 23, 293. 10.3892/mmr.2021.11933 33649775 PMC7931006

[B87] Fang S. Xu H. Lu J. Zhu Y. Jiang H. (2013). Neuroprotection by the Kappa-Opioid Receptor Agonist, BRL52537, Is Mediated via Up-Regulating Phosphorylated Signal Transducer and Activator of Transcription-3 in Cerebral Ischemia/reperfusion Injury in Rats. Neurochem. Res. 38, 2305–2312. 10.1007/s11064-013-1139-4 23996400

[B88] Fantegrossi W. E. Kugle K. M. 3Rd Valdes L. J. Koreeda M. Woods J. H. (2005). Kappa-opioid Receptor-Mediated Effects of the Plant-Derived Hallucinogen, Salvinorin A, on Inverted Screen Performance in the Mouse. Behav. Pharmacol. 16, 627–633. 10.1097/00008877-200512000-00005 16286814

[B89] Fardin V. Plau B. Carruette A. Guyon C. Bardon T. Taurand G. (1990). RP 60180 : A Novel Phenothiazine with High Affinity for Kappa Binding Sites and with Antinociceptive Effects in Rodents, Pain 41, 192.

[B90] Fichna J. Dicay M. Lewellyn K. Janecka A. Zjawiony J. K. Macnaughton W. K. (2012). Salvinorin A Has Antiinflammatory and Antinociceptive Effects in Experimental Models of Colitis in Mice Mediated by KOR and CB1 Receptors. Inflamm. Bowel Dis. 18, 1137–1145. 10.1002/ibd.21873 21953882

[B91] Field M. J. Carnell A. J. Gonzalez M. I. Mccleary S. Oles R. J. Smith R. (1999). Enadoline, a Selective Kappa-Opioid Receptor Agonist Shows Potent Antihyperalgesic and Antiallodynic Actions in a Rat Model of Surgical Pain. Pain 80, 383–389. 10.1016/s0304-3959(98)00237-1 10204752

[B92] Finley M. J. Chen X. Bardi G. Davey P. Geller E. B. Zhang L. (2008). Bi-directional Heterologous Desensitization between the Major HIV-1 Co-receptor CXCR4 and the Kappa-Opioid Receptor. J. Neuroimmunol. 197, 114–123. 10.1016/j.jneuroim.2008.04.021 18533278 PMC2596309

[B93] Fishbane S. Mathur V. Germain M. J. Shirazian S. Bhaduri S. Munera C. (2020). Randomized Controlled Trial of Difelikefalin for Chronic Pruritus in Hemodialysis Patients. Kidney Int. Rep. 5, 600–610. 10.1016/j.ekir.2020.01.006 32405581 PMC7210745

[B94] Foster J. S. Moore R. N. (1987). Dynorphin and Related Opioid Peptides Enhance Tumoricidal Activity Mediated by Murine Peritoneal Macrophages. J. Leukoc. Biol. 42, 171–174. 10.1002/jlb.42.2.171 2439627

[B95] Fountas A. Chai S. T. Kourkouti C. Karavitaki N. (2018). Mechanisms of Endocrinology: Endocrinology of Opioids. Eur. J. Endocrinol. 179, R183–R196. 10.1530/EJE-18-0270 30299887

[B96] Fraitag B. Homerin M. Hecketsweiler P. (1994). Double-blind Dose-Response Multicenter Comparison of Fedotozine and Placebo in Treatment of Nonulcer Dyspepsia. Dig. Dis. Sci. 39, 1072–1077. 10.1007/BF02087560 8174419

[B97] France C. P. Medzihradsky F. Woods J. H. (1994). Comparison of Kappa Opioids in Rhesus Monkeys: Behavioral Effects and Receptor Binding Affinities. J. Pharmacol. Exp. Ther. 268, 47–58. 8301589

[B98] Frankowski K. J. Hedrick M. P. Gosalia P. Li K. Shi S. Whipple D. (2012). Discovery of Small Molecule Kappa Opioid Receptor Agonist and Antagonist Chemotypes through a HTS and Hit Refinement Strategy. ACS Chem. Neurosci. 3, 221–236. 10.1021/cn200128x 22737280 PMC3378255

[B99] Gadano A. Moreau R. Pessione F. Trombino C. Giuily N. Sinnassamy P. (2000). Aquaretic Effects of Niravoline, a Kappa-Opioid Agonist, in Patients with Cirrhosis. J. Hepatol. 32, 38–42. 10.1016/s0168-8278(00)80187-7 10673065

[B100] Gardell L. R. Spencer R. H. Chalmers D. T. Al E. (2008). Preclinical Profile of CR845: A Novel, Long-Acting Peripheral Kappa Opioid Receptor Agonist International Association for the Study of Pain 2008.

[B101] Gavériaux-Ruff C. Simonin F. Filliol D. Kieffer B. L. (2003). Enhanced Humoral Response in Kappa-Opioid Receptor Knockout Mice. J. Neuroimmunol. 134, 72–81. 10.1016/s0165-5728(02)00419-8 12507774

[B102] Giardina G. Clarke G. D. Dondio G. Petrone G. Sbacchi M. Vecchietti V. (1994). Selective Kappa-Opioid Agonists: Synthesis and Structure-Activity Relationships of Piperidines Incorporating on Oxo-Containing Acyl Group. J. Med. Chem. 37, 3482–3491. 10.1021/jm00047a006 7932577

[B103] Gillis A. Kliewer A. Kelly E. Henderson G. Christie M. J. Schulz S. (2020). Critical Assessment of G Protein-Biased Agonism at the μ-Opioid Receptor. Trends Pharmacol. Sci. 41, 947–959. 10.1016/j.tips.2020.09.009 33097283

[B104] Giros B. Pohl M, Rochelle J. M. Seldin M. F. (1995). Chromosomal Localization of Opioid Peptide and Receptor Genes in the Mouse. Life Sci. 56, PL369–75. 10.1016/0024-3205(95)00119-q 7752808

[B105] Glue P. Lockhart M. Lam F. Hung N. Hung C. T. Friedhoff L. (2015). Ascending-dose Study of Noribogaine in Healthy Volunteers: Pharmacokinetics, Pharmacodynamics, Safety, and Tolerability. J. Clin. Pharmacol. 55, 189–194. 10.1002/jcph.404 25279818

[B106] Gmerek D. E. Cowan A. (1984). *In Vivo* evidence for Benzomorphan-Selective Receptors in Rats. J. Pharmacol. Exp. Ther. 230, 110–115. 6146703

[B107] Goldberg D. S. McGee S. J. (2011). Pain as a Global Public Health Priority. BMC Public Health 11, 770. 10.1186/1471-2458-11-770 21978149 PMC3201926

[B108] Goldblatt F. Isenberg D. A. (2005). New Therapies for Rheumatoid Arthritis. Clin. Exp. Immunol. 140, 195–204. 10.1111/j.1365-2249.2005.02744.x 15807842 PMC1809355

[B109] Gottshall S. L. Cassel J. A. Cortes Burgos L. Daubert J. D. Dehaven R. N. Koblish M. (1999). Antinociception Produced by ADL 10-0101, a Novel Peripheral K Opioid Receptor Agonist, in Models of Inflammatory Hyperalgesia. Soc. Neurosci. Abstr. 25, 1221.

[B110] Goyagi T. Toung T. J. Kirsch J. R. Traystman R. J. Koehler R. C. Hurn P. D. (2003). Neuroprotective Kappa-Opioid Receptor Agonist BRL 52537 Attenuates Ischemia-Evoked Nitric Oxide Production *In Vivo* in Rats. Stroke 34, 1533–1538. 10.1161/01.STR.0000072512.30658.E7 12738895

[B111] Guéniau C. Oberlander C. (1997). The Kappa Opioid Agonist Niravoline Decreases Brain Edema in the Mouse Middle Cerebral Artery Occlusion Model of Stroke. J. Pharmacol. Exp. Ther. 282, 1–6. 9223533

[B112] Gunji N. Nagashima M. Asano G. Yoshino S. (2000). Expression of Kappa-Opioid Receptor mRNA in Human Peripheral Blood Lymphocytes and the Relationship between its Expression and the Inflammatory Changes in Rheumatoid Arthritis. Rheumatol. Int. 19, 95–100. 10.1007/s002960050110 10776687

[B113] Günther T. Dasgupta P. Mann A. Miess E. Kliewer A. Fritzwanker S. (2018). Targeting Multiple Opioid Receptors - Improved Analgesics with Reduced Side Effects? Br. J. Pharmacol. 175, 2857–2868. 10.1111/bph.13809 28378462 PMC6016677

[B114] Gupta A. Gomes I. Bobeck E. N. Fakira A. K. Massaro N. P. Sharma I. (2016). Collybolide Is a Novel Biased Agonist of κ-opioid Receptors with Potent Antipruritic Activity. Proc. Natl. Acad. Sci. U. S. A. 113, 6041–6046. 10.1073/pnas.1521825113 27162327 PMC4889365

[B115] Haber S. N. Kowall N. W. Vonsattel J. P. Bird E. D. Richardson E. P. (1986). Gilles de la Tourette's syndrome. A postmortem neuropathological and immunohistochemical study. J. Neurol. Sci. 75, 225–241. 10.1016/0022-510x(86)90097-3 2428943

[B116] Hägermark Ö. Hökfelt T. Pernow B. (1978). Flare and Itch Induced by Substance P in Human Skin. J. Invest. Dermatol 71 (4). 10.1111/1523-1747.ep12515092 81243

[B117] Hauser S. L. Cree B. A. C. (2020). Treatment of Multiple Sclerosis: A Review. Am. J. Med. 133, 1380–e2. 10.1016/j.amjmed.2020.05.049 32682869 PMC7704606

[B118] Hayes A. G. Tyers M. B. (1983). Determination of Receptors that Mediate Opiate Side Effects in the Mouse. Br. J. Pharmacol. 79, 731–736. 10.1111/j.1476-5381.1983.tb10011.x 6317119 PMC2044905

[B119] Hojjati S. M. Zarghami A. Hojjati S. A. Baes M. (2015). Optic Neuritis, the Most Common Initial Presenting Manifestation of Multiple Sclerosis in Northern Iran. Casp. J. Intern Med. 6, 151–155. PMC4650790 26644882

[B120] Holzer P. (2009). Opioid Receptors in the Gastrointestinal Tract. Regul. Pept. 155, 11–17. 10.1016/j.regpep.2009.03.012 19345246 PMC3163293

[B121] Horan P. de Costa B. R. Rice K. C. Porreca F. (1991). Differential Antagonism of U69,593- and Bremazocine-Induced Antinociception by (-)-UPHIT: Evidence of Kappa Opioid Receptor Multiplicity in Mice. J. Pharmacol. Exp. Ther. 257, 1154–1161. 1646325

[B122] Hoskin P. J. Hanks G. W. (1991). Opioid Agonist-Antagonist Drugs in Acute and Chronic Pain States. Drugs 41, 326–344. 10.2165/00003495-199141030-00002 1711441

[B123] Hughes F. M. JR. Shaner B. E. Brower J. O. Woods R. J. Dix T. A. (2013). Development of a Peptide-Derived Orally-Active Kappa-Opioid Receptor Agonist Targeting Peripheral Pain. Open Med. Chem. J. 7, 16–22. 10.2174/1874104501307010016 24222801 PMC3821081

[B124] Hughes N. R. Mcknight A. T. Woodruff G. N. Hill M. P. Crossman A. R. Brotchie J. M. (1998). Kappa-opioid Receptor Agonists Increase Locomotor Activity in the Monoamine-Depleted Rat Model of Parkinsonism. Mov. Disord. 13, 228–233. 10.1002/mds.870130206 9539334

[B125] Huskinson S. L. Platt D. M. Brasfield M. Follett M. E. Prisinzano T. E. Blough B. E. (2020). Quantification of Observable Behaviors Induced by Typical and Atypical Kappa-Opioid Receptor Agonists in Male Rhesus Monkeys. Psychopharmacol. Berl. 237, 2075–2087. 10.1007/s00213-020-05519-7 PMC7308209 32372348

[B126] Ignatowski T. A. Bidlack J. M. (1998). Detection of Kappa Opioid Receptors on Mouse Thymocyte Phenotypic Subpopulations as Assessed by Flow Cytometry. J. Pharmacol. Exp. Ther. 284, 298–306. 9435191

[B127] Ikeda K. Yoshikawa S. Kurokawa T. Yuzawa N. Nakao K. Mochizuki H. (2009). TRK-820, a Selective Kappa Opioid Receptor Agonist, Could Effectively Ameliorate L-DOPA-Induced Dyskinesia Symptoms in a Rat Model of Parkinson's Disease. Eur. J. Pharmacol. 620, 42–48. 10.1016/j.ejphar.2009.08.013 19686730

[B128] Ikeda Y. Toda S. Kawamoto T. Teramoto A. (1997). Arginine Vasopressin Release Inhibitor RU51599 Attenuates Brain Oedema Following Transient Forebrain Ischaemia in Rats. Acta Neurochir. (Wien) 139, 1166–1172. 10.1007/BF01410978 9479424

[B129] Inan S. Cowan A. (2004). Kappa Opioid Agonists Suppress Chloroquine-Induced Scratching in Mice. Eur. J. Pharmacol. 502, 233–237. 10.1016/j.ejphar.2004.09.010 15476749

[B130] Inan S. Dun N. J. Cowan A. (2009). Nalfurafine Prevents 5'-guanidinonaltrindole- and Compound 48/80-induced Spinal C-Fos Expression and Attenuates 5'-Guanidinonaltrindole-Elicited Scratching Behavior in Mice. Neuroscience 163, 23–33. 10.1016/j.neuroscience.2009.06.016 19524022 PMC2735087

[B131] Inan S. Torres-Huerta A. Jensen L. E. Dun N. J. Cowan A. (2019). Nalbuphine, a Kappa Opioid Receptor Agonist and Mu Opioid Receptor Antagonist Attenuates Pruritus, Decreases IL-31, and Increases IL-10 in Mice with Contact Dermatitis. Eur. J. Pharmacol. 864, 172702. 10.1016/j.ejphar.2019.172702 31568781 PMC6913640

[B132] Institute of Medicine (2011). Relieving Pain in America: A Blueprint for Transforming Prevention, Care, Education, and Research. Washington, D.C.: Institute of Medicine. 10.7205/MILMED-D-16-00012 27136641

[B133] Ishihara K. Hirano T. (2002). IL-6 in Autoimmune Disease and Chronic Inflammatory Proliferative Disease. Cytokine Growth Factor Rev. 13, 357–368. 10.1016/s1359-6101(02)00027-8 12220549

[B134] Iwaszkiewicz K. S. Schneider J. J. Hua S. (2013). Targeting Peripheral Opioid Receptors to Promote Analgesic and Anti-inflammatory Actions. Front. Pharmacol. 4, 132. 10.3389/fphar.2013.00132 24167491 PMC3807052

[B135] Jamshidi R. J. Jacobs B. A. Sullivan L. C. Chavera T. A. Saylor R. M. Prisinzano T. E. (2015). Functional Selectivity of Kappa Opioid Receptor Agonists in Peripheral Sensory Neurons. J. Pharmacol. Exp. Ther. 355, 174–182. 10.1124/jpet.115.225896 26297384 PMC4613959

[B136] Ji H. Wang Y. Liu G. Xu X. Dai D. Chen Z. (2015). OPRK1 Promoter Hypermethylation Increases the Risk of Alzheimer's Disease. Neurosci. Lett. 606, 24–29. 10.1016/j.neulet.2015.08.027 26300544

[B137] Jin-Cheng L. Wen Y. Zhao Y. Quan-Yu Z. Shu-Miao Z. Hai-Tao G. (2008). Anti-arrhythmic Effects of Kappa-Opioid Receptor and its Changes in Ischemia and Reperfusion. Arch. Med. Res. 39, 483–488. 10.1016/j.arcmed.2008.02.011 18514092

[B138] Jung Y. H. Kim Y. O. Han J. H. Kim Y. C. Yoon M. H. (2017). Isobolographic Analysis of Drug Combinations with Intrathecal BRL52537 (κ-Opioid Agonist), Pregabalin (Calcium Channel Modulator), AF 353 (P2X3 Receptor Antagonist), and A804598 (P2X7 Receptor Antagonist) in Neuropathic Rats. Anesth. Analg. 125, 670–677. 10.1213/ANE.0000000000001883 28277328

[B139] Kale N. (2016). Optic Neuritis as an Early Sign of Multiple Sclerosis. Eye Brain 8, 195–202. 10.2147/EB.S54131 28539814 PMC5398757

[B140] Kamei J. Nagase H. (2001). Norbinaltorphimine, a Selective Kappa-Opioid Receptor Antagonist, Induces an Itch-Associated Response in Mice. Eur. J. Pharmacol. 418, 141–145. 10.1016/s0014-2999(01)00941-4 11334876

[B141] Kapusta D. R. Obih J. C. (1993). Central Kappa Opioid Receptor-Evoked Changes in Renal Function in Conscious Rats: Participation of Renal Nerves. J. Pharmacol. Exp. Ther. 267, 197–204. 8229746

[B142] Karussis D. (2014). The Diagnosis of Multiple Sclerosis and the Various Related Demyelinating Syndromes: a Critical Review. J. Autoimmun. 48-49, 134–142. 10.1016/j.jaut.2014.01.022 24524923

[B143] Kaski S. W. White A. N. Gross J. D. Trexler K. R. Wix K. Harland A. A. (2019). Preclinical Testing of Nalfurafine as an Opioid-Sparing Adjuvant that Potentiates Analgesia by the Mu Opioid Receptor-Targeting Agonist Morphine. J. Pharmacol. Exp. Ther. 371, 487–499. 10.1124/jpet.118.255661 31492823 PMC6863463

[B144] Keïta H. Kayser V. Guilbaud G. (1995). Antinociceptive Effect of a κ-opioid Receptor Agonist that Minimally Crosses the Blood-Brain Barrier (ICI 204448) in a Rat Model of Mononeuropathy. Eur. J. Pharmacol. 277, 275–280. 7493620 10.1016/0014-2999(95)00122-2

[B145] Kieffer B. L. (1995). Recent Advances in Molecular Recognition and Signal Transduction of Active Peptides: Receptors for Opioid Peptides. Cell Mol. Neurobiol. 15, 615–635. 10.1007/BF02071128 8719033 PMC11563145

[B146] Kivell B. Prisinzano T. E. (2010). Kappa Opioids and the Modulation of Pain. Psychopharmacol. Berl. 210, 109–119. 10.1007/s00213-010-1819-6 20372880

[B147] Kivell B. M. Paton K. F. Kumar N. Morani A. S. Culverhouse A. Shepherd A. (2018). Kappa Opioid Receptor Agonist Mesyl Sal B Attenuates Behavioral Sensitization to Cocaine with Fewer Aversive Side-Effects Than Salvinorin A in Rodents. Molecules 23. 10.3390/molecules23102602 PMC6222496 30314288

[B148] Knoll A. T. Carlezon W. A. JR. (2010). Dynorphin, Stress, and Depression. Brain Res. 1314, 56–73. 10.1016/j.brainres.2009.09.074 19782055 PMC2819644

[B149] Ko M.-C. Husbands S. M. (2020). Pleiotropic Effects of Kappa Opioid Receptor-Related Ligands in Non-human Primates. Handb. Exp. Pharmacol. 271, 435–452. 10.1007/164_2020_419 PMC8175454 33274403

[B150] Ko M. C. Husbands S. M. (2009). Effects of Atypical Kappa-Opioid Receptor Agonists on Intrathecal Morphine-Induced Itch and Analgesia in Primates. J. Pharmacol. Exp. Ther. 328, 193–200. 10.1124/jpet.108.143925 18842704 PMC2719014

[B151] Ko M. C. (2015). Neuraxial Opioid-Induced Itch and its Pharmacological Antagonism. Handb. Exp. Pharmacol. 226, 315–335. 10.1007/978-3-662-44605-8_17 25861787 PMC4447088

[B152] Kögel B. Christoph T. Friderichs E. Hennies H. H. Matthiesen T. Schneider J. (1998). HZ2, a Selective Kappa-Opioid Agonist. CNS Drug Rev. 4, 54–70.

[B153] Kozono H. Yoshitani H. Nakano R. (2018). Post-marketing Surveillance Study of the Safety and Efficacy of Nalfurafine Hydrochloride (Remitch® Capsules 2.5 μg) in 3,762 Hemodialysis Patients with Intractable Pruritus. Int. J. Nephrol. Renov. Dis. 11, 9–24. 10.2147/IJNRD.S145720 PMC5774492 29391822

[B154] Krajnc N. Berger T. Bsteh G. (2021). Measuring Treatment Response in Progressive Multiple Sclerosis-Considerations for Adapting to an Era of Multiple Treatment Options. Biomolecules 11, 1342. 10.3390/biom11091342 34572555 PMC8470215

[B155] Kuhar M. J. Pert C. B. Snyder S. H. (1973). Regional Distribution of Opiate Receptor Binding in Monkey and Human Brain. Nature 245, 447–450. 10.1038/245447a0 4127185

[B156] Kumagai H. Ebata T. Takamori K. Muramatsu T. Nakamoto H. Suzuki H. (2010). Effect of a Novel Kappa-Receptor Agonist, Nalfurafine Hydrochloride, on Severe Itch in 337 Haemodialysis Patients: a Phase III, Randomized, Double-Blind, Placebo-Controlled Study. Nephrol. Dial. Transpl. 25, 1251–1257. 10.1093/ndt/gfp588 19926718

[B157] Kuzmin A. Sandin J. Terenius L. Ogren S. O. (2000). Dose- and Time-dependent Bimodal Effects of Kappa-Opioid Agonists on Locomotor Activity in Mice. J. Pharmacol. Exp. Ther. 295, 1031–1042. 11082438

[B158] Kuzumaki N. Suzuki A. Narita M. Hosoya T. Nagasawa A. Imai S. (2012). Effect of κ-opioid Receptor Agonist on the Growth of Non-small Cell Lung Cancer (NSCLC) Cells. Br. J. Cancer 106, 1148–1152. 10.1038/bjc.2011.574 22343623 PMC3304401

[B159] La Regina A. Petrillo P. Sbacchi M. Tavani A. (1988). Interaction of U-69,593 with Mu-, Alpha- and Kappa-Opioid Binding Sites and its Analgesic and Intestinal Effects in Rats. Life Sci. 42, 293–301. 10.1016/0024-3205(88)90638-8 2826959

[B160] Land B. B. Bruchas M. R. Lemos J. C. Xu M. Melief E. J. Chavkin C. (2008). The Dysphoric Component of Stress Is Encoded by Activation of the Dynorphin Kappa-Opioid System. J. Neurosci. 28, 407–414. 10.1523/JNEUROSCI.4458-07.2008 18184783 PMC2612708

[B161] Leander J. D. (1983). A Kappa Opioid Effect: Increased Urination in the Rat. J. Pharmacol. Exp. Ther. 224, 89–94. 6294284

[B162] Li X. Sun Y. Jin Q. Song D. Diao Y. (2019). Kappa Opioid Receptor Agonists Improve Postoperative Cognitive Dysfunction in Rats via the JAK2/STAT3 Signaling Pathway. Int. J. Mol. Med. 44, 1866–1876. 10.3892/ijmm.2019.4339 31545485 PMC6777679

[B163] Lin J. Wang H. Li J. Wang Q. Zhang S. Feng N. (2013). κ-Opioid Receptor Stimulation Modulates TLR4/NF-Κb Signaling in the Rat Heart Subjected to Ischemia-Reperfusion. Cytokine 61, 842–848. 10.1016/j.cyto.2013.01.002 23402995

[B164] Lipman Z. M. Yosipovitch G. (2021). An Evaluation of Difelikefalin as a Treatment Option for Moderate-To-Severe Pruritus in End Stage Renal Disease. Expert Opin. Pharmacother. 22, 549–555. 10.1080/14656566.2020.1849142 33190563

[B165] Liu J. J. Chiu Y. T. Dimattio K. M. Chen C. Huang P. Gentile T. A. (2019). Phosphoproteomic Approach for Agonist-specific Signaling in Mouse Brains: mTOR Pathway Is Involved in κ Opioid Aversion. Neuropsychopharmacology 44, 939–949. 10.1038/s41386-018-0155-0 30082888 PMC6462019

[B166] Long J. M. Holtzman D. M. (2019). Alzheimer Disease: an Update on Pathobiology and Treatment Strategies. Cell 179, 312–339. 10.1016/j.cell.2019.09.001 31564456 PMC6778042

[B167] Lötsch J. Ditterich W. Hummel T. Kobal G. (1997). Antinociceptive Effects of the Kappa-Opioid Receptor Agonist RP 60180 Compared with Pentazocine in an Experimental Human Pain Model. Clin. Neuropharmacol. 20, 224–233. 10.1097/00002826-199706000-00006 9197945

[B168] Lovell K. M. Frankowski K. J. Stahl E. L. Slauson S. R. Yoo E. Prisinzano T. E. (2015). Structure-activity Relationship Studies of Functionally Selective Kappa Opioid Receptor Agonists that Modulate ERK 1/2 Phosphorylation while Preserving G Protein over βarrestin2 Signaling Bias. ACS Chem. Neurosci. 6, 1411–1419. 10.1021/acschemneuro.5b00092 25891774 PMC4830356

[B169] Machelska H. Celik M. Ö. (2018). Advances in Achieving Opioid Analgesia without Side Effects. Front. Pharmacol. 9, 1388. 10.3389/fphar.2018.01388 30555325 PMC6282113

[B170] MacLean K. A. Johnson M. W. Reissig C. J. Prisinzano T. E. Griffiths R. R. (2013). Dose-related Effects of Salvinorin A in Humans: Dissociative, Hallucinogenic, and Memory Effects. Psychopharmacol. Berl. 226, 381–392. 10.1007/s00213-012-2912-9 PMC3581702 23135605

[B171] Mangel A. W. Bornstein J. D. Hamm L. R. Buda J. Wang J. Irish W. (2008). Clinical Trial: Asimadoline in the Treatment of Patients with Irritable Bowel Syndrome. Aliment. Pharmacol. Ther. 28, 239–249. 10.1111/j.1365-2036.2008.03730.x 18466359

[B172] Mansour A. Fox C. A. Akil H. Watson S. J. (1995). Opioid-receptor mRNA Expression in the Rat CNS: Anatomical and Functional Implications. Trends Neurosci. 18, 22–29. 10.1016/0166-2236(95)93946-u 7535487

[B173] Maqueda A. E. Valle M. Addy P. H. Antonijoan R. M. Puntes M. Coimbra J. (2015). Salvinorin-A Induces Intense Dissociative Effects, Blocking External Sensory Perception and Modulating Interoception and Sense of Body Ownership in Humans. Int. J. Neuropsychopharmacol. 18. 10.1093/ijnp/pyv065 PMC4675976 26047623

[B174] Marin C. Bové J. Bonastre M. Tolosa E. (2003). Effect of Acute and Chronic Administration of U50,488, a Kappa Opioid Receptor Agonist, in 6-OHDA-Lesioned Rats Chronically Treated with Levodopa. Exp. Neurol. 183, 66–73. 10.1016/s0014-4886(03)00107-9 12957489

[B175] Marsella R. Ahrens K. Wilkes R. Soeberdt M. Abels C. (2021). Topical κ-opioid Receptor Agonist Asimadoline Improves Dermatitis in a Canine Model of Atopic Dermatitis. Exp. Dermatol 31 (4), 628–632. 10.1111/exd.14507 34839557

[B176] Mathieu-Kia A.-M. Fan L.-Q. Kreek M. J. Simon E. J. Hiller J. M. (2001). Μ-, δ- and κ-opioid Receptor Populations Are Differentially Altered in Distinct Areas of Postmortem Brains of Alzheimer's Disease Patients. Brain Res. 893, 121–134. 10.1016/s0006-8993(00)03302-3 11223000

[B177] Mathur V. S. Kumar J. Crawford P. W. Hait H. Sciascia T. (2017). A Multicenter, Randomized, Double-Blind, Placebo-Controlled Trial of Nalbuphine ER Tablets for Uremic Pruritus. Am. J. Nephrol. 46, 450–458. 10.1159/000484573 29253847

[B178] Matthes H. W. Maldonado R. Simonin F. Valverde O. Slowe S. Kitchen I. (1996). Loss of Morphine-Induced Analgesia, Reward Effect and Withdrawal Symptoms in Mice Lacking the Mu-Opioid-Receptor Gene. Nature 383, 819–823. 10.1038/383819a0 8893006

[B179] McCurdy C. R. Sufka K. J. Smith G. H. Warnick J. E. Nieto M. J. (2006). Antinociceptive Profile of Salvinorin A, a Structurally Unique Kappa Opioid Receptor Agonist. Pharmacol. Biochem. Behav. 83, 109–113. 10.1016/j.pbb.2005.12.011 16434091

[B180] Meariman J. Sutphen J. Gao J. Kapusta D. R. (2021). Nalfurafine, an Orally Active, G Protein-Biased Kappa Opioid Receptor Agonist, Increases the Diuretic Response to Loop and Thiazide Diuretics and Limits Electrolyte Losses. Circulation 144, A13956. 10.1161/HYPERTENSIONAHA.121.18503

[B181] Mei F. Mayoral S. R. Nobuta H. Wang F. Desponts C. Lorrain D. S. (2016). Identification of the Kappa-Opioid Receptor as a Therapeutic Target for Oligodendrocyte Remyelination. J. Neurosci. 36, 7925–7935. 10.1523/JNEUROSCI.1493-16.2016 27466337 PMC4961778

[B182] Melnicoff M. J. Horan P. K. Morahan P. S. (1989). Kinetics of Changes in Peritoneal Cell Populations Following Acute Inflammation. Cell Immunol. 118, 178–191. 10.1016/0008-8749(89)90367-5 2910501

[B183] Ménard C. Herzog H. Schwarzer C. Quirion R. (2014). Possible Role of Dynorphins in Alzheimer's Disease and Age-Related Cognitive Deficits. Neurodegener. Dis. 13, 82–85. 10.1159/000353848 23970097

[B184] Mercadante S. Romualdi P. (2020). The Therapeutic Potential of Novel Kappa Opioid Receptor-Based Treatments. Curr. Med. Chem. 27, 2012–2020. 10.2174/0929867326666190121142459 30666905

[B185] Morani A. S. Kivell B. Prisinzano T. E. Schenk S. (2009). Effect of Kappa-Opioid Receptor Agonists U69593, U50488H, Spiradoline and Salvinorin A on Cocaine-Induced Drug-Seeking in Rats. Pharmacol. Biochem. Behav. 94, 244–249. 10.1016/j.pbb.2009.09.002 19747933 PMC3021564

[B186] Moreau R. Cailmail S. Hamon G. Lebrec D. (1996). Renal and Haemodynamic Responses to a Novel Kappa Opioid Receptor Agonist, Niravoline (RU 51,599), in Rats with Cirrhosis. J. Gastroenterol. Hepatol. 11, 857–863. 10.1111/j.1440-1746.1996.tb00093.x 8889966

[B187] Mores K. L. Cummins B. R. Cassell R. J. van Rijn R. M. (2019). A Review of the Therapeutic Potential of Recently Developed G Protein-Biased Kappa Agonists. Front. Pharmacol. 10, 407. 10.3389/fphar.2019.00407 31057409 PMC6478756

[B188] Morgenweck J. Frankowski K. J. Prisinzano T. E. Aubé J. Bohn L. M. (2015). Investigation of the Role of βarrestin2 in Kappa Opioid Receptor Modulation in a Mouse Model of Pruritus. Neuropharmacology 99, 600–609. 10.1016/j.neuropharm.2015.08.027 26318102 PMC4739521

[B189] Muratspahić E. Tomašević N. Nasrollahi-Shirazi S. Gattringer J. Emser F. S. Freissmuth M. (2021). Plant-Derived Cyclotides Modulate κ-Opioid Receptor Signaling. J. Nat. Prod. 84, 2238–2248. 10.1021/acs.jnatprod.1c00301 34308635 PMC8406418

[B190] Muto M. Mori M. Sato Y. Uzawa A. Masuda S. Uchida T. (2015). Current Symptomatology in Multiple Sclerosis and Neuromyelitis Optica. Eur. J. Neurol. 22, 299–304. 10.1111/ene.12566 25264295

[B191] Nagase H. Hayakawa J. Kawamura K. Kawai K. Takezawa Y. Matsuura H. (1998). Discovery of a Structurally Novel Opioid Kappa-Agonist Derived from 4,5-epoxymorphinan. Chem. Pharm. Bull. (Tokyo) 46, 366–369. 10.1248/cpb.46.366 9501472

[B192] Nakao K. Mochizuki H. (2009). Nalfurafine Hydrochloride: a New Drug for the Treatment of Uremic Pruritus in Hemodialysis Patients. Drugs Today (Barc) 45, 323–329. 10.1358/dot.2009.45.5.1362067 19584962

[B193] Nakasone T. Sato T. Matsushima Y. Inoue T. Kamei C. (2015). Characteristics of Scratching Behavior in ADJM Mice (Atopic Dermatitis from Japanese Mice). Immunopharmacol. Immunotoxicol. 37, 202–206. 10.3109/08923973.2014.1001903 25578901

[B194] Nemeth C. L. Paine T. A. Rittiner J. E. Béguin C. Carroll F. I. Roth B. L. (2010). Role of Kappa-Opioid Receptors in the Effects of Salvinorin A and Ketamine on Attention in Rats. Psychopharmacol. Berl. 210, 263–274. 10.1007/s00213-010-1834-7 PMC2869248 20358363

[B195] Nestby P. Schoffelmeer A. N. Homberg J. R. Wardeh G. De Vries T. J. Mulder A. H. (1999). Bremazocine Reduces Unrestricted Free-Choice Ethanol Self-Administration in Rats without Affecting Sucrose Preference. Psychopharmacol. Berl. 142, 309–317. 10.1007/s002130050894 10208324

[B196] Obara I. Parkitna J. R. Korostynski M. Makuch W. Kaminska D. Przewlocka B. (2009). Local Peripheral Opioid Effects and Expression of Opioid Genes in the Spinal Cord and Dorsal Root Ganglia in Neuropathic and Inflammatory Pain. Pain 141, 283–291. 10.1016/j.pain.2008.12.006 19147290

[B197] Pandey S. Srivanitchapoom P. (2017). Levodopa-induced Dyskinesia: Clinical Features, Pathophysiology, and Medical Management. Ann. Indian Acad. Neurol. 20, 190–198. 10.4103/aian.AIAN_239_17 28904447 PMC5586110

[B198] Paris J. J. Reilley K. J. Mclaughlin J. P. (2011). Kappa Opioid Receptor-Mediated Disruption of Novel Object Recognition: Relevance for Psychostimulant Treatment. J. Addict. Res. Ther. S4. 10.4172/2155-6105.S4-007 PMC3418884 22900234

[B199] Parkhill A. L. Bidlack J. M. (2006). Reduction of Lipopolysaccharide-Induced Interleukin-6 Production by the Kappa Opioid U50,488 in a Mouse Monocyte-like Cell Line. Int. Immunopharmacol. 6, 1013–1019. 10.1016/j.intimp.2006.01.012 16644488

[B200] Pasternak G. W. Pan Y. X. (2013). Mu Opioids and Their Receptors: Evolution of a Concept. Pharmacol. Rev. 65, 1257–1317. 10.1124/pr.112.007138 24076545 PMC3799236

[B201] Paton K. F. Atigari D. V. Kaska S. Prisinzano T. Kivell B. M. (2020a). Strategies for Developing κ Opioid Receptor Agonists for the Treatment of Pain with Fewer Side Effects. J. Pharmacol. Exp. Ther. 375, 332–348. 10.1124/jpet.120.000134 32913006 PMC7589957

[B202] Paton K. F. Biggerstaff A. Kaska S. Crowley R. S. La Flamme A. C. Prisinzano T. E. (2020b). Evaluation of Biased and Balanced Salvinorin A Analogs in Preclinical Models of Pain. Front. Neurosci. 14, 765. 10.3389/fnins.2020.00765 32792903 PMC7385413

[B203] Paton K. F. Kumar N. Crowley R. S. Harper J. L. Prisinzano T. E. Kivell B. M. (2017). The Analgesic and Anti-inflammatory Effects of Salvinorin A Analogue β-tetrahydropyran Salvinorin B in Mice. Eur. J. Pain 21, 1039–1050. 10.1002/ejp.1002 28158929 PMC5466480

[B204] Paton K. F. Robichon K. Templeton N. Denny L. Abadey A. A. Luo D. (2021). The Salvinorin Analogue, Ethoxymethyl Ether Salvinorin B, Promotes Remyelination in Preclinical Models of Multiple Sclerosis. Front. Neurology 12. 10.3389/fneur.2021.782190 PMC8721439 34987466

[B205] Pattinson K. T. (2008). Opioids and the Control of Respiration. Br. J. Anaesth. 100, 747–758. 10.1093/bja/aen094 18456641

[B206] Peart J. N. Gross E. R. Gross G. J. (2004). Effect of Exogenous Kappa-Opioid Receptor Activation in Rat Model of Myocardial Infarction. J. Cardiovasc Pharmacol. 43, 410–415. 10.1097/00005344-200403000-00012 15076225

[B207] Peckys D. Landwehrmeyer G. B. (1999). Expression of Mu, Kappa, and Delta Opioid Receptor Messenger RNA in the Human CNS: a 33P *In Situ* Hybridization Study. Neuroscience 88, 1093–1135. 10.1016/s0306-4522(98)00251-6 10336124

[B208] Peng J. Sarkar S. Chang S. L. (2012). Opioid Receptor Expression in Human Brain and Peripheral Tissues Using Absolute Quantitative Real-Time RT-PCR. Drug Alcohol Depend. 124, 223–228. 10.1016/j.drugalcdep.2012.01.013 22356890 PMC3366045

[B209] Peters G. R. Ward N. J. Antal E. G. Lai P. Y. Demaar E. W. (1987). Diuretic Actions in Man of a Selective Kappa Opioid Agonist: U-62,066E. J. Pharmacol. Exp. Ther. 240, 128–131. 3027300

[B210] Prisinzano T. E. (2005). Psychopharmacology of the Hallucinogenic Sage Salvia Divinorum. Life Sci. 78, 527–531. 10.1016/j.lfs.2005.09.008 16213533

[B211] Privette T. H. Terrian D. M. (1995). Kappa Opioid Agonists Produce Anxiolytic-like Behavior on the Elevated Plus-Maze. Psychopharmacol. Berl. 118, 444–450. 10.1007/BF02245945 7568631

[B212] Pugsley M. K. Saint D. A. Hayes E. S. Kramer D. Walker M. J. (1998). Sodium Channel-Blocking Properties of Spiradoline, a Kappa Receptor Agonist, Are Responsible for its Antiarrhythmic Action in the Rat. J. Cardiovasc Pharmacol. 32, 863–874. 10.1097/00005344-199812000-00002 9869491

[B213] Quirion B. Bergeron F. Blais V. Gendron L. (2020). The Delta-Opioid Receptor; a Target for the Treatment of Pain. Front. Mol. Neurosci. 13, 52. 10.3389/fnmol.2020.00052 32431594 PMC7214757

[B214] Ranganathan M. Schnakenberg A. Skosnik P. D. Cohen B. M. Pittman B. Sewell R. A. (2012). Dose-related Behavioral, Subjective, Endocrine, and Psychophysiological Effects of the κ Opioid Agonist Salvinorin A in Humans. Biol. Psychiatry 72, 871–879. 10.1016/j.biopsych.2012.06.012 22817868 PMC3638802

[B215] Rawls S. M. Ding Z. Gray A. M. Cowan A. (2005). Peripheral Kappa-Opioid Agonist, ICI 204448, Evokes Hypothermia in Cold-Exposed Rats. Pharmacology 74, 79–83. 10.1159/000083704 15687734

[B216] Read N. W. Abitbol J. L. Bardhan K. D. Whorwell P. J. Fraitag B. (1997). Efficacy and Safety of the Peripheral Kappa Agonist Fedotozine versus Placebo in the Treatment of Functional Dyspepsia. Gut 41, 664–668. 10.1136/gut.41.5.664 9414975 PMC1891553

[B217] Rezvani A. Mash D. Hearn W. Lee Y. Overstreet D. (1995). Noribogaine, a Primary Ibogaine Metabolite, Reduces Alcohol Intake in P and Fawn-Hooded Rats. Alcohol Clin. Exp. Res. 19, 15A.

[B218] Riviere P. J. Pascaud X. Chevalier E. Junien J. L. (1994). Fedotozine Reversal of Peritoneal-Irritation-Induced Ileus in Rats: Possible Peripheral Action on Sensory Afferents. J. Pharmacol. Exp. Ther. 270, 846–850. 7932195

[B219] Rogers T. J. (2020). Bidirectional Regulation of Opioid and Chemokine Function. Front. Immunol. 11, 94. 10.3389/fimmu.2020.00094 32076421 PMC7006827

[B220] Rommer P. S. Eichstädt K. Ellenberger D. Flachenecker P. Friede T. Haas J. (2019). Symptomatology and Symptomatic Treatment in Multiple Sclerosis: Results from a Nationwide MS Registry. Mult. Scler. 25, 1641–1652. 10.1177/1352458518799580 30230952

[B221] Rong F. Peng Z. Ye M. X. Zhang Q. Y. Zhao Y. Zhang S. M. (2009). Myocardial Apoptosis and Infarction after Ischemia/reperfusion Are Attenuated by Kappa-Opioid Receptor Agonist. Arch. Med. Res. 40, 227–234. 10.1016/j.arcmed.2009.04.009 19608010

[B222] Rowbotham M. C. Wallace M. (2020). Evolution of Analgesic Tolerance and Opioid-Induced Hyperalgesia over 6 Months: Double-Blind Randomized Trial Incorporating Experimental Pain Models. J. Pain 21, 1031–1046. 10.1016/j.jpain.2020.01.005 32006699

[B223] Salaga M. Mokrowiecka A. Jacenik D. Cygankiewicz A. I. Malecka-Panas E. Kordek R. (2017). Systemic Administration of Sialorphin Attenuates Experimental Colitis in Mice via Interaction with Mu and Kappa Opioid Receptors. J. Crohns Colitis 11, 988–998. 10.1093/ecco-jcc/jjx043 28333341

[B224] Sałaga M. Polepally P. R. Sobczak M. Grzywacz D. Kamysz W. Sibaev A. (2014). Novel Orally Available Salvinorin A Analog PR-38 Inhibits Gastrointestinal Motility and Reduces Abdominal Pain in Mouse Models Mimicking Irritable Bowel Syndrome. J. Pharmacol. Exp. Ther. 350, 69–78. 10.1124/jpet.114.214239 24891526

[B225] Salas S. P. Roblero J. Ureta H. Huidobro-Toro J. P. (1989). Diuretic Effect of Bremazocine, a Kappa-Opioid with Central and Peripheral Sites of Action. J. Pharmacol. Exp. Ther. 250, 992–999. 2550625

[B226] Salemi S. Aeschlimann A. Reisch N. Jüngel A. Gay R. E. Heppner F. L. (2005). Detection of Kappa and Delta Opioid Receptors in Skin-Ooutside the Nervous System. Biochem. Biophys. Res. Commun. 338, 1012–1017. 10.1016/j.bbrc.2005.10.072 16263089

[B227] Sandner-Kiesling A. Pan H. L. Chen R. L. James R. L. Dehaven-Hudkins D. L. Dewan D. M. (2002). Effect of Kappa Opioid Agonists on Visceral Nociception Induced by Uterine Cervical Distension in Rats. Pain 96, 13–22. 10.1016/s0304-3959(01)00398-0 11932057

[B228] Scharf J. M. Miller L. L. Gauvin C. A. Alabiso J. Mathews C. A. Ben-Shlomo Y. (2015). Population Prevalence of Tourette Syndrome: a Systematic Review and Meta-Analysis. Mov. Disord. 30, 221–228. 10.1002/mds.26089 25487709

[B229] Schemann M. Kugler E. M. Buhner S. Eastwood C. Donovan J. Jiang W. (2012). The Mast Cell Degranulator Compound 48/80 Directly Activates Neurons. PLoS One 7, e52104. 10.1371/journal.pone.0052104 23272218 PMC3525567

[B230] Sengupta J. N. Snider A. Su X. Gebhart G. F. (1999). Effects of Kappa Opioids in the Inflamed Rat Colon. Pain 79, 175–185. 10.1016/s0304-3959(98)00175-4 10068163

[B231] Shahbazian A. Heinemann A. Schmidhammer H. Beubler E. Holzer-Petsche U. Holzer P. (2002). Involvement of Mu- and Kappa-, but Not Delta-, Opioid Receptors in the Peristaltic Motor Depression Caused by Endogenous and Exogenous Opioids in the guinea-pig Intestine. Br. J. Pharmacol. 135, 741–750. 10.1038/sj.bjp.0704527 11834622 PMC1573189

[B232] Sharp B. M. Keane W. F. Suh H. J. Gekker G. Tsukayama D. Peterson P. K. (1985). Opioid Peptides Rapidly Stimulate Superoxide Production by Human Polymorphonuclear Leukocytes and Macrophages. Endocrinology 117, 793–795. 10.1210/endo-117-2-793 2862014

[B233] Shibata S. Tominaga K. Watanabe S. (1995). Kappa-Opioid Receptor Agonist Protects against Ischemic Reduction of 2-deoxyglucose Uptake in Morphine-Tolerant Rats. Eur. J. Pharmacol. 279, 197–202. 10.1016/0014-2999(95)00152-b 7556401

[B234] Silvia R. C. Slizgi G. R. Ludens J. H. Tang A. H. (1987). Protection from Ischemia-Induced Cerebral Edema in the Rat by U-50488H, a Kappa Opioid Receptor Agonist. Brain Res. 403, 52–57. 10.1016/0006-8993(87)90121-1 3030502

[B235] Simonson B. Morani A. S. Ewald A. W. Walker L. Kumar N. Simpson D. (2015). Pharmacology and Anti-addiction Effects of the Novel κ Opioid Receptor Agonist Mesyl Sal B, a Potent and Long-Acting Analogue of Salvinorin A. Br. J. Pharmacol. 172, 515–531. 10.1111/bph.12692 24641310 PMC4292965

[B236] Sobanski P. Krajnik M. Shaqura M. Bloch-Boguslawska E. Schäfer M. Mousa S. A. (2014). The Presence of Mu-, Delta-, and Kappa-Opioid Receptors in Human Heart Tissue. Heart Vessels 29, 855–863. 10.1007/s00380-013-0456-5 24390763

[B237] Sobczak M. Zakrzewski P. K. Cygankiewicz A. I. Mokrowiecka A. Chen C. Sałaga M. (2014). Anti-inflammatory Action of a Novel Orally Available Peptide 317 in Mouse Models of Inflammatory Bowel Diseases. Pharmacol. Rep. 66, 741–750. 10.1016/j.pharep.2014.03.007 25149976

[B238] Song X. Cui Z. He J. Yang T. Sun X. (2021). κ-Opioid Receptor Agonist, U50488H, Inhibits Pyroptosis through NLRP3 via the Ca(2+)/CaMKII/CREB Signaling Pathway and Improves Synaptic Plasticity in APP/PS1 Mice. Mol. Med. Rep. 24, 529. 10.3892/mmr.2021.12168 34036389 PMC8170177

[B239] Soulard C. D. Guerif S. Payne A. Dahl S. G. (1996). Differential Effects of Fedotozine Compared to Other Kappa Agonists on Diuresis in Rats. J. Pharmacol. Exp. Ther. 279, 1379–1385. 8968362

[B240] Sounvoravong S. Takahashi M. Nakashima M. N. Nakashima K. (2004). Disability of Development of Tolerance to Morphine and U-50,488H, a Selective Kappa-Opioid Receptor Agonist, in Neuropathic Pain Model Mice. J. Pharmacol. Sci. 94, 305–312. 10.1254/jphs.94.305 15037816

[B241] Spetea M. Eans S. O. Ganno M. L. Lantero A. Mairegger M. Toll L. (2017). Selective κ Receptor Partial Agonist HS666 Produces Potent Antinociception without Inducing Aversion after i.c.V. Administration in Mice. Br. J. Pharmacol. 174, 2444–2456. 10.1111/bph.13854 28494108 PMC5513865

[B242] Stein C. Millan M. J. Shippenberg T. S. Peter K. Herz A. (1989). Peripheral Opioid Receptors Mediating Antinociception in Inflammation. Evidence for Involvement of Mu, Delta and Kappa Receptors. J. Pharmacol. Exp. Ther. 248, 1269–1275. 2539460

[B243] Stutzmann J. M. Valin A Fau - Böhme G. A. Böhme Ga Fau - Fardin V. Fardin V Fau - Garret C. Garret C Fau - Naquet R. Naquet R. (1995). Electrographic Studies of the Effects of RP 60180, a Novel Kappa Agonist, on the Photosensitive Baboon *Papio papio* . Prog. Neuropsychopharmacol. Biol. Psychiatry 19 (4), 687–697. 10.1016/0278-5846(95)00112-9 8588066

[B244] Suzuki S. Chuang L. F. Doi R. H. Bidlack J. M. Chuang R. Y. (2001). *Kappa*-opioid Receptors on Lymphocytes of a Human Lymphocytic Cell Line: Morphine-Induced Up-Regulation as Evidenced by Competitive RT-PCR and Indirect Immunofluorescence. Int. Immunopharmacol. 1, 1733–1742. 10.1016/s1567-5769(01)00083-2 11562065

[B245] Suzuki T. Shiozaki Y. Masukawa Y. Misawa M. Nagase H. (1992). The Role of Mu- and Kappa-Opioid Receptors in Cocaine-Induced Conditioned Place Preference. Jpn. J. Pharmacol. 58, 435–442. 10.1254/jjp.58.435 1328733

[B246] Suzuki T. Tsuji M. Mori T. Misawa M. Endoh T. Nagase H. (1996). Effect of the Highly Selective and Nonpeptide Delta Opioid Receptor Agonist TAN-67 on the Morphine-Induced Place Preference in Mice. J. Pharmacol. Exp. Ther. 279, 177–185. 8858991

[B247] Takahashi K. Nakagawasai O. Sugawara M. Sato A. Nemoto W. Tadano T. (2018). Kappa Opioid Receptor Agonist Administration in Olfactory Bulbectomized Mice Restores Cognitive Impairment through Cholinergic Neuron Activation. Biol. Pharm. Bull. 41, 957–960. 10.1248/bpb.b18-00115 29863085

[B248] Tangherlini G. Börgel F. Schepmann D. Slocum S. Che T. Wagner S. (2020). Synthesis and Pharmacological Evaluation of Fluorinated Quinoxaline-Based κ-Opioid Receptor (KOR) Agonists Designed for PET Studies. ChemMedChem 15, 1834–1853. 10.1002/cmdc.202000502 33448685 PMC7589326

[B249] Tangherlini G. Kalinin D. V. Schepmann D. Che T. Mykicki N. Ständer S. (2019). Development of Novel Quinoxaline-Based κ-Opioid Receptor Agonists for the Treatment of Neuroinflammation. J. Med. Chem. 62, 893–907. 10.1021/acs.jmedchem.8b01609 30543421

[B250] Thell K. Hellinger R. Sahin E. Michenthaler P. Gold-Binder M. Haider T. (2016). Oral Activity of a Nature-Derived Cyclic Peptide for the Treatment of Multiple Sclerosis. Proc. Natl. Acad. Sci. U. S. A. 113, 3960–3965. 10.1073/pnas.1519960113 27035952 PMC4839460

[B251] Togashi Y. Umeuchi H. Okano K. Ando N. Yoshizawa Y. Honda T. (2002). Antipruritic Activity of the Kappa-Opioid Receptor Agonist, TRK-820. Eur. J. Pharmacol. 435, 259–264. 10.1016/s0014-2999(01)01588-6 11821035

[B252] Tominaga M. Ogawa H. Takamori K. (2007). Possible Roles of Epidermal Opioid Systems in Pruritus of Atopic Dermatitis. J. Invest. Dermatol 127, 2228–2235. 10.1038/sj.jid.5700942 17611580

[B253] Tong G. Zhang B. Zhou X. Zhao J. Sun Z. Tao Y. (2016). Kappa-opioid Agonist U50,488H-Mediated Protection against Heart Failure Following Myocardial Ischemia/reperfusion: Dual Roles of Heme Oxygenase-1. Cell Physiol. Biochem. 39, 2158–2172. 10.1159/000447911 27802429

[B254] Tortella F. C. Rose J. Robles L. Moreton J. E. Hughes J. Hunter J. C. (1997). EEG Spectral Analysis of the Neuroprotective Kappa Opioids Enadoline and PD117302. J. Pharmacol. Exp. Ther. 282, 286–293. 9223566

[B255] Tosk J. M. Grim J. R. Kinback K. M. Sale E. J. Bozzetti L. P. Will A. D. (1993). Modulation of Chemiluminescence in a Murine Macrophage Cell Line by Neuroendocrine Hormones. Int. J. Immunopharmacol. 15, 615–620. 10.1016/0192-0561(93)90079-e 8104166

[B256] Umeuchi H. Togashi Y. Honda T. Nakao K. Okano K. Tanaka T. (2003). Involvement of Central Mu-Opioid System in the Scratching Behavior in Mice, and the Suppression of it by the Activation of Kappa-Opioid System. Eur. J. Pharmacol. 477, 29–35. 10.1016/j.ejphar.2003.08.007 14512095

[B257] Uprety R. Che T. Zaidi S. A. Grinnell S. G. Varga B. R. Faouzi A. (2021). Controlling Opioid Receptor Functional Selectivity by Targeting Distinct Subpockets of the Orthosteric Site. Elife 10, e56519. 10.7554/eLife.56519 33555255 PMC7909954

[B258] Ur E. Wright D. M. Bouloux P. M. Grossman A. (1997). The Effects of Spiradoline (U-62066E), a Kappa-Opioid Receptor Agonist, on Neuroendocrine Function in Man. Br. J. Pharmacol. 120, 781–784. 10.1038/sj.bjp.0700971 9138682 PMC1564535

[B259] Valentino R. J. Volkow N. D. (2018). Untangling the Complexity of Opioid Receptor Function. Neuropsychopharmacology 43, 2514–2520. 10.1038/s41386-018-0225-3 30250308 PMC6224460

[B260] Valenza M. Butelman E. R. Kreek M. J. (2017). "Effects of the Novel Relatively Short-Acting Kappa Opioid Receptor Antagonist LY2444296 in Behaviors Observed after Chronic Extended-Access Cocaine Self-Administration in Rats". Psychopharmacol. Berl. 234, 2219–2231. 10.1007/s00213-017-4647-0 PMC5591939 28550455

[B261] van der Knaap M. S. Bugiani M. (2017). Leukodystrophies: a Proposed Classification System Based on Pathological Changes and Pathogenetic Mechanisms. Acta Neuropathol. 134, 351–382. 10.1007/s00401-017-1739-1 28638987 PMC5563342

[B262] Vanderah T. W. Largent-Milnes T. Lai J. Porreca F. Houghten R. A. Menzaghi F. (2008). Novel D-Amino Acid Tetrapeptides Produce Potent Antinociception by Selectively Acting at Peripheral Kappa-Opioid Receptors. Eur. J. Pharmacol. 583, 62–72. 10.1016/j.ejphar.2008.01.011 18282565

[B263] Viscusi E. R. Torjman M. C. Munera C. L. Stauffer J. W. Setnik B. S. Bagal S. N. (2021). Effect of Difelikefalin, a Selective Kappa Opioid Receptor Agonist, on Respiratory Depression: A Randomized, Double-Blind, Placebo-Controlled Trial. Clin. Transl. Sci. 14, 1886–1893. 10.1111/cts.13042 33982405 PMC8504812

[B264] Von voigtlander P. F. Lahti R. A. Ludens J. H. (1983). U-50,488: a Selective and Structurally Novel Non-mu (Kappa) Opioid Agonist. J. Pharmacol. Exp. Ther. 224, 7–12. 6129321

[B265] Von Voigtlander P. F. Lewis R. A. (1982). U-50,488, a Selective Kappa Opioid Agonist: Comparison to Other Reputed Kappa Agonists. Prog. Neuropsychopharmacol. Biol. Psychiatry 6, 467–470. 10.1016/s0278-5846(82)80130-9 6298890

[B266] Vuong C. van Uum S. H. O'Dell L. E. Lutfy K. Friedman T. C. (2010). The Effects of Opioids and Opioid Analogs on Animal and Human Endocrine Systems. Endocr. Rev. 31, 98–132. 10.1210/er.2009-0009 19903933 PMC2852206

[B267] Wadenberg M. L. (2003). A Review of the Properties of Spiradoline: a Potent and Selective Kappa-Opioid Receptor Agonist. CNS Drug Rev. 9, 187–198. 10.1111/j.1527-3458.2003.tb00248.x 12847558 PMC6741666

[B268] Walker J. S. Howlett C. R. Nayanar V. (1995). Anti-inflammatory Effects of Kappa-Opioids in Adjuvant Arthritis. Life Sci. 57, 371–378. 10.1016/0024-3205(95)00296-i 7603309

[B269] Walsh S. L. Strain E. C. Abreu M. E. Bigelow G. E. (2001). Enadoline, a Selective Kappa Opioid Agonist: Comparison with Butorphanol and Hydromorphone in Humans. Psychopharmacol. Berl. 157, 151–162. 10.1007/s002130100788 11594439

[B270] Walton C. King R. Rechtman L. Kaye W. Leray E. Marrie R. A. (2020). Rising Prevalence of Multiple Sclerosis Worldwide: Insights from the Atlas of MS, Third Edition. Mult. Scler. 26, 1816–1821. 10.1177/1352458520970841 third edition 33174475 PMC7720355

[B271] Wang F. Yang Y. J. Yang N. Chen X. J. Huang N. X. Zhang J. (2018). Enhancing Oligodendrocyte Myelination Rescues Synaptic Loss and Improves Functional Recovery after Chronic Hypoxia. Neuron 99, 689–e5. e5. 10.1016/j.neuron.2018.07.017 30078577 PMC6170028

[B272] Wang X. Gou X. Yu X. Bai D. Tan B. Cao P. (2021). Antinociceptive and Antipruritic Effects of HSK21542, a Peripherally-Restricted Kappa Opioid Receptor Agonist, in Animal Models of Pain and Itch. Front. Pharmacol. 12, 773204. 10.3389/fphar.2021.773204 34867403 PMC8635029

[B273] Wang Y. Chen Y. Xu W. Lee D. Y. Ma Z. Rawls S. M. (2008). 2-Methoxymethyl-salvinorin B Is a Potent Kappa Opioid Receptor Agonist with Longer Lasting Action *In Vivo* Than Salvinorin A. J. Pharmacol. Exp. Ther. 324, 1073–1083. 10.1124/jpet.107.132142 18089845 PMC2519046

[B274] Wang Y. Tang K. Inan S. Siebert D. Holzgrabe U. Lee D. Y. (2005). Comparison of Pharmacological Activities of Three Distinct Kappa Ligands (Salvinorin A, TRK-820 and 3FLB) on Kappa Opioid Receptors *In Vitro* and Their Antipruritic and Antinociceptive Activities *In Vivo* . J. Pharmacol. Exp. Ther. 312, 220–230. 10.1124/jpet.104.073668 15383632

[B275] Weber A. E. Jalali O. Limfat S. Shkhyan R. van der Horst R. Lee S. (2020). Modulation of Hedgehog Signaling by Kappa Opioids to Attenuate Osteoarthritis. Arthritis Rheumatol. 72, 1278–1288. 10.1002/art.41250 32249508 PMC7541630

[B276] Wee S. Koob G. F. (2010). The Role of the Dynorphin-Kappa Opioid System in the Reinforcing Effects of Drugs of Abuse. Psychopharmacol. Berl. 210, 121–135. 10.1007/s00213-010-1825-8 PMC2879894 20352414

[B277] Wei Y.-Y. Ma Y. Yao S.-Y. Kong L.-H. Liu X. Chai J.-R. (2021). Novel Selective κ Agonists SLL-039 and SLL-1206 Produce Potent Antinociception with Fewer Sedation and Aversion. Acta Pharmacol. Sin. 10.1038/s41401-021-00761-x PMC9160296 34493813

[B278] Weidinger S. Beck L. A. Bieber T. Kabashima K. Irvine A. D. (2018). Atopic Dermatitis. Nat. Rev. Dis. Prim. 4, 1. 10.1038/s41572-018-0001-z 29930242

[B279] Wilson J. L. Nayanar V. Walker J. S. (1996). The Site of Anti-arthritic Action of the Kappa-Opioid, U-50, 488H, in Adjuvant Arthritis: Importance of Local Administration. Br. J. Pharmacol. 118, 1754–1760. 10.1111/j.1476-5381.1996.tb15601.x 8842441 PMC1909829

[B280] Wong W. (2020). Economic Burden of Alzheimer Disease and Managed Care Considerations. Am. J. Manag. Care 26, S177–S183. 10.37765/ajmc.2020.88482 32840331

[B281] Wu L. Zhang S. Shkhyan R. Lee S. Gullo F. Eliasberg C. D. (2017). Kappa Opioid Receptor Signaling Protects Cartilage Tissue against Posttraumatic Degeneration. JCI Insight 2, e88553–S442. 10.1172/jci.insight.88553 28097228 PMC5214705

[B282] Wu T. Yao H. Zhang B. Zhou S. Hou P. Chen K. (2021). κ Opioid Receptor Agonist Inhibits Myocardial Injury in Heart Failure Rats through Activating Nrf2/HO-1 Pathway and Regulating Ca2+-SERCA2a. Oxid. Med. Cell Longev. 2021, 7328437. 10.1155/2021/7328437 34373768 PMC8349291

[B283] Wu X. Zhang B. Fan R. Zhao L. Wang Y. Zhang S. (2011). U50,488H Inhibits Neutrophil Accumulation and TNF-α Induction Induced by Ischemia-Reperfusion in Rat Heart. Cytokine 56, 503–507. 10.1016/j.cyto.2011.07.015 21843951

[B284] Xin J. Zhang Y. He Z. Wang Z. (2016). Highly Selective Non-opioid Kappa Opioid Receptor (KOR) Agonist Salvinorin A Protects against Forebrain Ischemia-Induced Brain Injury in Rats. Brain Res. 1637, 168–176. 10.1016/j.brainres.2016.02.024 26907190

[B285] Yamada K. Imai M. Yoshida S. (1989). Mechanism of Diuretic Action of U-62,066E, a Kappa Opioid Receptor Agonist. Eur. J. Pharmacol. 160, 229–237. 10.1016/0014-2999(89)90495-0 2547626

[B286] Yamamizu K. Furuta S. Hamada Y. Yamashita A. Kuzumaki N. Narita M. (2013). К Opioids Inhibit Tumor Angiogenesis by Suppressing VEGF Signaling. Sci. Rep. 3, 3213. 10.1038/srep03213 PMC3827603 24225480

[B287] Yamamizu K. Furuta S. Katayama S. Narita M. Kuzumaki N. Imai S. (2011). The κ Opioid System Regulates Endothelial Cell Differentiation and Pathfinding in Vascular Development. Blood 118, 775–785. 10.1182/blood-2010-09-306001 21460241

[B288] Yiannopoulou K. G. Papageorgiou S. G. (2020). Current and Future Treatments in Alzheimer Disease: an Update. J. Cent. Nerv. Syst. Dis. 12, 1179573520907397. 10.1177/1179573520907397 32165850 PMC7050025

[B289] Young N. P. Weinshenker B. G. Parisi J. E. Scheithauer B. Giannini C. Roemer S. F. (2010). Perivenous Demyelination: Association with Clinically Defined Acute Disseminated Encephalomyelitis and Comparison with Pathologically Confirmed Multiple Sclerosis. Brain 133, 333–348. 10.1093/brain/awp321 20129932 PMC2822631

[B290] Zamarripa C. A. Pareek T. Schrock H. M. Prisinzano T. E. Blough B. E. Sufka K. J. (2021). The Kappa-Opioid Receptor Agonist, Triazole 1.1, Reduces Oxycodone Self-Administration and Enhances Oxycodone-Induced Thermal Antinociception in Male Rats. Psychopharmacol. Berl. 238, 3463–3476. 10.1007/s00213-021-05965-x PMC8629928 34430992

[B291] Zebraski S. E. Kochenash S. M. Raffa R. B. (2000). Lung Opioid Receptors: Pharmacology and Possible Target for Nebulized Morphine in Dyspnea. Life Sci. 66, 2221–2231. 10.1016/s0024-3205(00)00434-3 10855942

[B292] Zelaya C. A. Dahlhamer J. M. Lucas J. W. Connor E. M. (2020). Chronic Pain and High-Impact Chronic Pain Among u.S. Adults, 2019. NCHS Data Brief., 1–8. 33151145

[B293] Zhang L. S. Wang J. Chen J. C. Tao Y. M. Wang Y. H. Xu X. J. (2015). Novel κ-opioid Receptor Agonist MB-1C-OH Produces Potent Analgesia with Less Depression and Sedation. Acta Pharmacol. Sin. 36, 565–571. 10.1038/aps.2014.145 25816912 PMC4422940

[B294] Zhang Y. Butelman E. R. Schlussman S. D. Ho A. Kreek M. J. (2005). Effects of the Plant-Derived Hallucinogen Salvinorin A on Basal Dopamine Levels in the Caudate Putamen and in a Conditioned Place Aversion Assay in Mice: Agonist Actions at Kappa Opioid Receptors. Psychopharmacol. Berl. 179, 551–558. 10.1007/s00213-004-2087-0 15682306

[B295] Zhou L. Lovell K. M. Frankowski K. J. Slauson S. R. Phillips A. M. Streicher J. M. (2013). Development of Functionally Selective, Small Molecule Agonists at Kappa Opioid Receptors. J. Biol. Chem. 288, 36703–36716. 10.1074/jbc.M113.504381 24187130 PMC3868780

[B296] Zhou Q. Zhang Z. Long S. Li W. Wang B. Liang N. (2022). Opioids in Cancer: The κ-opioid Receptor (Review). Mol. Med. Rep. 25, 44. 10.3892/mmr.2021.12560 34878160 PMC8674701

[B297] Zhou Y. Kreek M. J. (2019). Combination of Clinically Utilized Kappa-Opioid Receptor Agonist Nalfurafine with Low-Dose Naltrexone Reduces Excessive Alcohol Drinking in Male and Female Mice. Alcohol Clin. Exp. Res. 43, 1077–1090. 10.1111/acer.14033 30908671 PMC6551307

[B298] Zielińska M. Szymaszkiewicz A. Jacenik D. Schodel L. Sałaga M. Zatorski H. (2020). Cyclic Derivative of Morphiceptin Dmt-cyclo-(D-Lys-Phe-D-Pro-Asp)-NH2(P-317), a Mixed Agonist of MOP and KOP Opioid Receptors, Exerts Anti-inflammatory and Anti-tumor Activity in Colitis and Colitis-Associated Colorectal Cancer in Mice. Eur. J. Pharmacol. 885, 173463. 10.1016/j.ejphar.2020.173463 32835668

